# Cryo–electron microscopy structure and analysis of the P-Rex1–Gβγ signaling scaffold

**DOI:** 10.1126/sciadv.aax8855

**Published:** 2019-10-16

**Authors:** Jennifer N. Cash, Sarah Urata, Sheng Li, Sandeep K. Ravala, Larisa V. Avramova, Michael D. Shost, J. Silvio Gutkind, John J. G. Tesmer, Michael A. Cianfrocco

**Affiliations:** 1 Department of Biological Chemistry & Life Sciences Institute, University of Michigan, Ann Arbor, MI, USA.; 2 Department of Medicine, University of California, San Diego, San Diego, CA, USA.; 3 Departments of Biological Sciences and of Medicinal Chemistry and Molecular Pharmacology, Purdue University, West Lafayette, IN, USA.; 4 Department of Pharmacology and Moores Cancer Center, University of California, San Diego, San Diego, CA, USA.

## Abstract

PIP_3_-dependent Rac exchanger 1 (P-Rex1) is activated downstream of G protein–coupled receptors to promote neutrophil migration and metastasis. The structure of more than half of the enzyme and its regulatory G protein binding site are unknown. Our 3.2 Å cryo-EM structure of the P-Rex1–Gβγ complex reveals that the carboxyl-terminal half of P-Rex1 adopts a complex fold most similar to those of *Legionella* phosphoinositide phosphatases. Although catalytically inert, the domain coalesces with a DEP domain and two PDZ domains to form an extensive docking site for Gβγ. Hydrogen-deuterium exchange mass spectrometry suggests that Gβγ binding induces allosteric changes in P-Rex1, but functional assays indicate that membrane localization is also required for full activation. Thus, a multidomain assembly is key to the regulation of P-Rex1 by Gβγ and the formation of a membrane-localized scaffold optimized for recruitment of other signaling proteins such as PKA and PTEN.

## Teaser

The cryo-EM structure of P-Rex1 bound to Gβγ reveals the configuration of an important membrane-associated signaling scaffold.

## INTRODUCTION

**Fig. 1:**
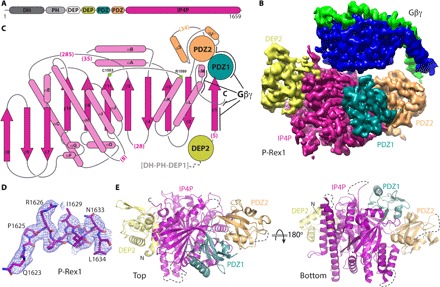
Cryo-EM structure of the P-Rex1–Gβγ complex reveals a complex Gβγ-binding module comprising four P-Rex1 domains. (**A**) Domain layout of P-Rex1. The domains shown in shades of gray were conformationally heterogeneous with respect to the Gβγ-binding module. (**B**) Cryo-EM map of the C-terminal 1153 residues of P-Rex1 in complex with Gβγ. (**C**) Topology diagram of the P-Rex1 IP4P domain. The DEP2 and PDZ domains are shown as circles and adopt canonical folds. The PDZ2 domain has two extra helices (α2´ and α3) and an additional β strand (β4′, not shown). Gβγ interaction sites are indicated with black lines. Parentheses indicate the number of unmodeled residues in loops. Green circles correspond to canonical phosphatase catalytic residues. (**D**) Example map density and fitted model from the IP4P domain. (**E**) “Top” and “bottom” views of P-Rex1 relative to (B) with Gβγ removed for clarity. Dashed lines represent disordered loops.

Phosphatidylinositol 3,4,5-trisphosphate (PIP_3_)–dependent Rac exchanger 1 (P-Rex1) plays key roles in neutrophil function and breast, prostate, and skin cancer metastasis (*1*–*6*) by activating small guanosine triphosphatases (GTPases) in response to extracellular signals. This 1659–amino acid Rho guanine nucleotide exchange factor (RhoGEF) (*7*) is activated downstream of G protein–coupled receptors and phosphoinositide-3-kinase via heterotrimeric G protein βγ (Gβγ) subunits and PIP_3_, respectively (*8*). The N-terminal half of the enzyme contains signaling domains with well-known folds, including the Dbl homology (DH) and pleckstrin homology (PH) domains characteristic of the Dbl family of RhoGEFs (*9*, *10*), two DEP domains, and two PDZ domains (Fig. 1A). However, the structure and function of its C-terminal half are poorly understood, and it contains a large amount of low-complexity sequence. This region displays weak sequence homology to inositol polyphosphate-4-phosphatase type I (IP4P) in that it has a canonical phosphatase C*X*_5_R active site signature motif (*11*) close to the C terminus. However, it has no detectable phosphatase activity (*8*).

Studies on the isolated PH domain in complex with the head group of PIP_3_ [Protein Data Bank (PDB): 5D3X and 5D3Y] confirmed that it is the primary PIP_3_ binding site necessary for P-Rex1 activation in cells (*9*, *12*). However, the location of the Gβγ-binding site and how its interaction contributes to P-Rex1 activation are unresolved (*12*–*14*). It has been reported that the GEF activity of the isolated P-Rex1 DH/PH domain tandem can be activated by Gβγ, as can the activity of a P-Rex1 variant lacking the PH domain, suggesting that Gβγ binds to the DH domain (*12*). However, the same study also showed that deleting just the IP4P domain eliminated activation by Gβγ, indicating that the IP4P domain is also involved in regulation by Gβγ. Activation of the isolated DH domain by Gβγ was also reported (*14*). In contrast, immunoprecipitation experiments showed that Gβγ binds to a tertiary structure formed by the IP4P domain along with the second DEP and first PDZ domains (*13*). Furthermore, some studies have shown that Gβγ can activate P-Rex1 on its own (*8*, *12*), whereas others indicated that PIP_3_ is required to observe Gβγ-mediated effects (*9*, *15*). Details in experimental differences may not only underline these discrepancies but also highlight our lack in understanding of the mechanisms controlling Gβγ activation of P-Rex1. To resolve the structure of the P-Rex1 IP4P domain, how this domain is organized with respect to other P-Rex1 domains, and the binding site for Gβγ, we used cryo-electron microscopy (cryo-EM) complemented by hydrogen-deuterium exchange mass spectrometry (HDX-MS) and functional enzymatic assays. Our results reveal that the principal binding site for Gβγ is at the C terminus of the IP4P domain and that the binding of Gβγ and PIP_3_ to P-Rex1 together invokes an allosteric change in addition to membrane recruitment to enhance P-Rex1 GEF activity.

## RESULTS

### Cryo-EM structure of the P-Rex1–Gβγ complex

**Fig. 2:**
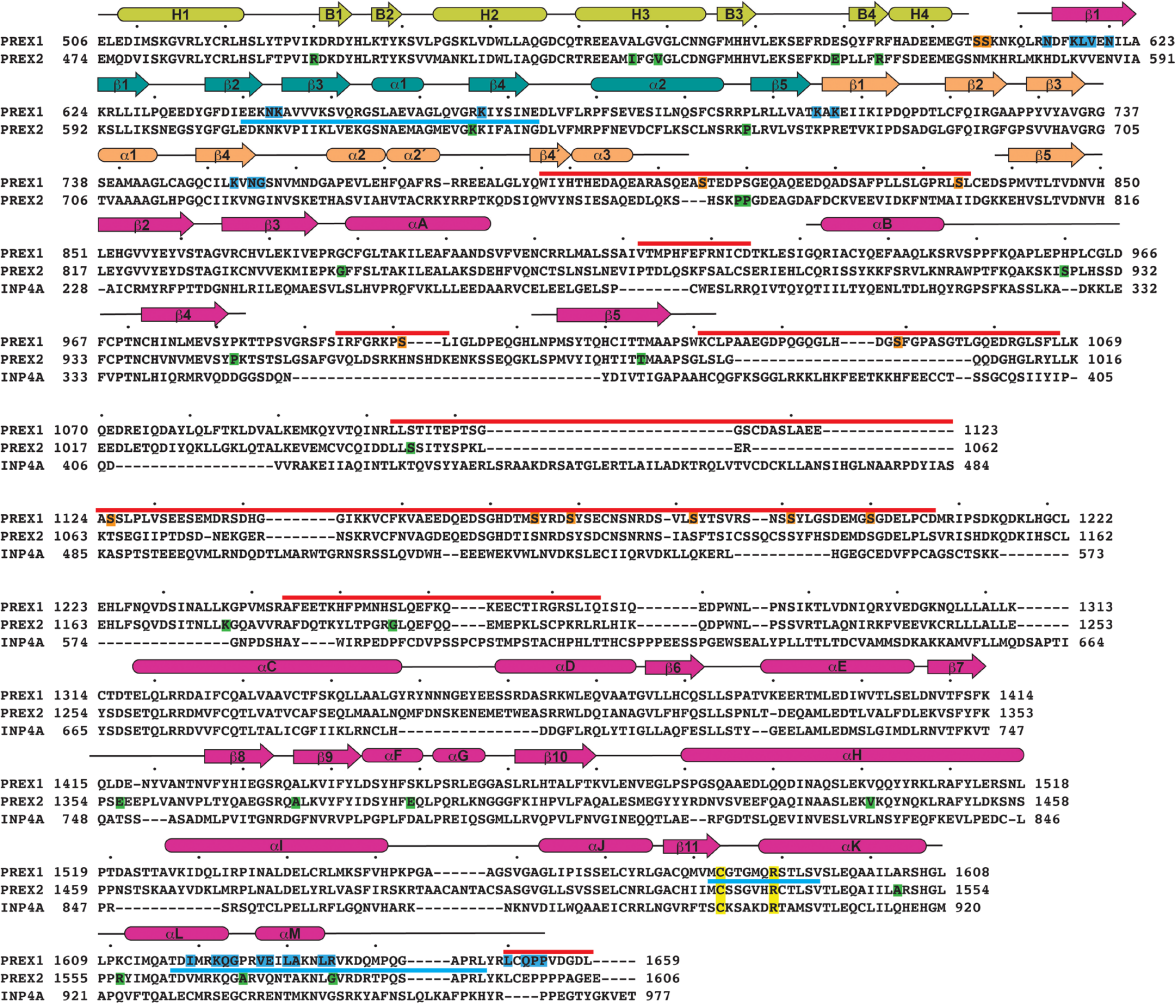
Sequence alignment of P-Rex1 with its close homologs along with other annotated characteristics. Although residues 38 to 505 of P-Rex1 were present in the protein used for cryo-EM analysis, they were not observed in the high-resolution reconstruction and are not shown here. Clustal Omega was used to align the sequences of human P-Rex1 (UniProtKB ID Q8TCU6), P-Rex2 (ID Q70Z35), and inositol 3,4-bisphosphate 4-phosphatase (INPP4A; ID Q96PE3). Residues 1 to 227 of INPP4A, corresponding to its N-terminal C2 domain, were excluded. The dots above the alignment correspond to every 10th amino acid in the P-Rex1 sequence. The secondary structure elements observed in P-Rex1 are shown above the alignment, with α helices depicted as rounded rectangles, β strands as arrows, and coil as a black line. They are colored by their corresponding domain as defined in Fig. 1. The absence of indicated secondary structure indicates that these residues were not observed in the structure. Thick red bars above the sequence correspond to P-Rex1 regions that are >90% exchanged with deuterium after 1000 s (see fig. S9 and data file S1). Thick blue bars indicate regions that are significantly stabilized (4% or greater protection at 1000 s) during HDX-MS in the presence of Gβγ (see Fig. 5 and data files S1 and S2). Residues highlighted in blue correspond to those that bury ≥5 Å^2^ of accessible surface area in the Gβγ complex (out of a total of 1000 Å^2^ buried accessible surface area on P-Rex1). Most also correspond to regions that are stabilized in HDX-MS upon complex formation. Residues highlighted in yellow correspond to canonical cysteine and arginine active site residues found in PTEN and *Legionella* phosphoinositide phosphatases SidF and SidP. Residues in P-Rex1 reported to be phosphorylated are highlighted in orange and are found in the more dynamic loops of the structure where protein kinases would have easier access. P-Rex2 residues that are associated with mutation in cancer patients are highlighted in green. Gβγ-binding residues are not conserved in INPP4A, and its phosphatase active site is much more basic than that of P-Rex1 in part due to the presence of two lysines in its P loop, analogous to those conserved in PTEN, SidF, and SidP, consistent with their robust phosphatase activity.

We used single-particle cryo-EM to determine the structure of nearly full-length human P-Rex1 in complex with Gβγ to 3.2 Å resolution. Initial attempts at determining this structure revealed a preferred orientation of the complex on cryo-EM grids, limiting its resolution to ~6 Å. We overcame this by collecting and merging together 0° and 30° tilted cryo-EM data to analyze ~900,000 particles and achieve a final structure with an overall resolution of 3.2 Å. This structure revealed a well-defined assembly of P-Rex1 domains spanning the C-terminal ~1100 amino acids of the protein (Figs. 1, B to E, and 2; figs. S1 to S3; and table S1). In addition, we observed two peripheral, low-resolution densities attributable to the N-terminal DH/PH-DEP1 domains and an unexpected domain most likely composed of elements within extended loops of the IP4P domain (fig. S4). The DEP2, PDZ1, and PDZ2 domains bury extensive surface area with the larger IP4P domain (Fig. 1E). DEP2 and PDZ1 (fig. S5) adopt canonical folds, but PDZ2 has additional secondary structural elements and a large disordered insertion (residues 804 to 837). Because the ~800-residue C-terminal IP4P domain of P-Rex1 had an unknown structure, we built it ab initio. Gβγ docks primarily with the IP4P domain but also makes contacts with both PDZ domains.

**Fig. 3:**
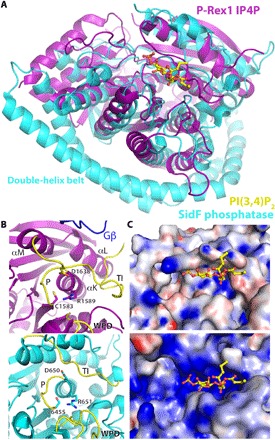
The P-Rex1 IP4P domain is a structural homolog of the *Legionella* phosphatase SidF. (**A**) IP4P domain superimposed on SidF (residues 183 to 743) in complex with PI(3,4)P_2_. For the figure, residues 1579 to 1589 of P-Rex1 were aligned with residues 641 to 651 of SidF. (**B**) Residues in P-Rex1 (top) compared with catalytic residues in SidF (bottom). A likely counterpart of Asp^650^ in SidF is Asp^1638^ on the TI loop of P-Rex1. Canonical phosphatase loops are indicated in yellow. A loop from Gβ is shown to indicate its proximity. (**C**) Surface representation colored by electrostatic potential of the structures shown in (B).

Unexpectedly, the C-terminal IP4P domain is structurally homologous to phosphoinositide 3-phosphatases from the bacterial genus *Legionella*, SidP (PDB: 4JZA) (*15*) and SidF (PDB: 4FYG) (*16*) (see Materials and Methods; Fig. 3A). The domain is an 11–β-strand elaboration of the canonical phosphatase fold, with strands β4, β5, β6, β10, and β11 corresponding to β1 to β5 in PTEN (PDB: 1D5R), respectively, with the canonical active site cysteine (*17*) located at the end of β11 (Figs. 1C and 2). The linker between DEP2 and PDZ1 forms the first strand of the core sheet (β1), fixing the position of these domains with respect to the IP4P domain (Fig. 1E). The β2 and β3 strands emerge immediately after PDZ2, and after two long helices, the structure begins the canonical phosphatase fold. The additional strands of the IP4P domain reside within a complex insertion between the β6 and β10 strands (Fig. 1C). The domain is decorated with large loops, including a 285-residue insertion between β5 and αC that is disordered in our structure. Considering these elements, the sequence homology to human INPP4A and INPP4B, phosphoinositide 4-phosphatases, becomes more obvious, although these proteins have N-terminal C2 domains instead of DH/PH, DEP, and PDZ domains (Fig. 2) (*11*).

### Analysis of the IP4P domain as a phosphatase

Like PTEN and other canonical phosphatases, the P-Rex1 IP4P domain bears the so-called TI, WPD, and catalytic C*X*_5_R P loops that form the active site (Fig. 3B) (*17*). Comparison of the P-Rex1 IP4P domain with the crystal structure of SidF bound to a substrate, PI(3,4)P_2_, suggests that P-Rex1 retains an intact phosphatase catalytic triad consisting of Cys^1583^, Arg^1589^, and Asp^1638^, although the final residue is found on the TI loop rather than on the P loop as in SidF (Fig. 3B). Although it has been reported that P-Rex1 is a pseudophosphatase (*8*), the existence and configuration of these residues compelled us to reevaluate the full-length P-Rex1 for Gβγ-dependent phosphatase activity because Gβγ interacts with elements on either end of the TI loop. However, comparing the activity of wild-type (WT) and C1583A P-Rex1 against a panel of potential phospholipid and phosphopeptide substrates did not reveal activity other than that attributed to contaminating phosphatases (fig. S6). Docking PI(3,4)P_2_ as bound to SidF into the vestigial catalytic site of P-Rex1 demonstrates that the TI loop partially occludes the pocket. Furthermore, the electrostatic surface potential of the pocket in P-Rex1 would not be complementary to the negative charge of PI substrates (Fig. 3C). Therefore, in the P-Rex1 IP4P domain, a structural element within a canonical phosphatase active site has been repurposed to form a Gβγ-binding site. It remains possible that P-Rex1 has phosphatase activity against as of yet unidentified substrates or in other signaling states.

### The interaction of P-Rex1 with Gβγ

**Fig. 4:**
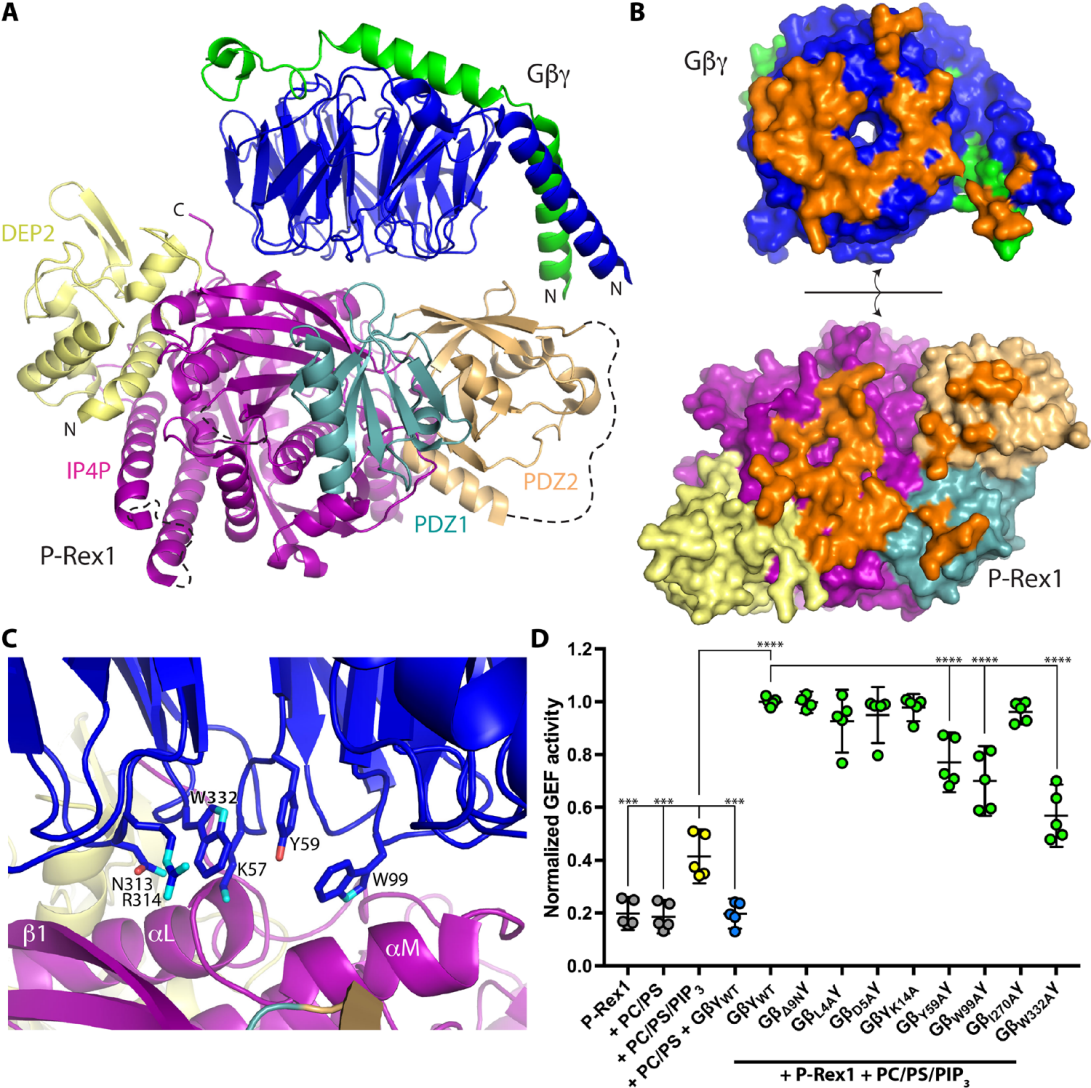
The P-Rex1 IP4P domain forms an extensive docking site for Gβγ. (**A**) Overview of Gβγ in complex with the P-Rex1 C-terminal Gβγ-binding module. (**B**) Surface representation of the complex in an “open book” view, with Gβγ and P-Rex1 peeled away from each other to visualize surfaces buried on each during complex formation (orange). (**C**) Close-up view of key interactions between Gβγ and the IP4P domain. (**D**) Liposome-based GEF assay demonstrating dependence of P-Rex1 activation on PIP_3_ and on Gβγ residues that interact with the IP4P domain. GEF activity in each experiment was normalized to that of WT Gβγ + PC/PS/PIP_3_.

Gβγ interacts with the IP4P domain (β1 strand and C terminus) and both PDZ domains of P-Rex1, burying ~2000 Å^2^ of accessible surface area (Fig. 4, A and B, and fig. S7). The P-Rex1 binding surface on Gβγ overlaps extensively with that used to bind Gα subunits (*18*) and other G protein effectors (*19*, *20*). A distinctive feature of the P-Rex1 complex, however, is that the N-terminal helices of the Gβ and Gγ chains also directly interact with P-Rex1, burying ~70 Å^2^ of solvent-accessible area on the PDZ2 domain (Fig. 4B and fig. S7A). The side chain of Gβ Trp^99^, involved in many Gβγ interactions, packs against the IP4P αM helix (Fig. 4C and fig. S7B). Other Gβ residues with large amounts of buried surface area include Leu^55^, which interacts with the P-Rex1 C terminus immediately after the TI loop, Gβ Lys^57^, which interacts with the αI-αM loop and the β1 strand, and Gβ Ile^270^, which interacts with a hairpin loop in PDZ1 (fig. S7C).

To assess the contributions of each P-Rex1 domain to Gβγ-mediated activation, we introduced mutations into Gβγ at each contact site and measured the ability of these variants to activate P-Rex1 in a liposome-based GEF assay with soluble Cdc42 as a substrate (Fig. 4D). PIP_3_ activated P-Rex1 nearly threefold with a half maximal effective concentration (EC_50_) of 1.6 μM (fig. S8A). WT Gβγ activated an additional two- to threefold with an EC_50_ of 44 nM (fig. S8B) but had no effect in the absence of PIP_3_ (Fig. 4D), consistent with some previous studies (*9*, *21*). Mutation of residues adjacent to the interface with PDZ2 (Gβ_L4A_, Gβ_D5A_, and Gγ_K14A_) or deleting the first nine amino acids of Gβ (Gβ_Δ9_) had no effect on GEF activity, suggesting that this interaction contributes little to activation in vitro. The Gβ_I270A_ mutation, disrupting the contact with PDZ1, likewise had no effect. Only the Gβ_Y59A_, Gβ_W99A_, and Gβ_W332A_ mutations, which perturb interactions with the IP4P domain, showed significant decreases in P-Rex1 activation (Fig. 4D). We analyzed the Gβ_W332A_γ variant further and showed that it exhibited a 10-fold higher EC_50_ value and lower efficacy than WT (fig. S8C). Thus, the interaction of Gβγ with the IP4P domain not only constitutes the majority of the buried surface area (Fig. 4B) but also is the principal driver of P-Rex1 activation in our assay. This result is consistent with a previous observation that truncation of the C-terminal 34 residues of P-Rex1 eliminates sensitivity to Gβγ (*13*).

### P-Rex1–Gβγ HDX-MS

Considering its prenylation, Gβγ could activate P-Rex1 via allosteric activation and/or membrane recruitment of P-Rex1. In our liposome-based GEF assay, soluble (C68S) Gβγ did not activate P-Rex1, even in the presence of PIP_3_ (fig. S8D), indicating that our Gβγ-mediated effects are dependent on the presence of a membrane. However, that does not preclude allosteric responses to Gβγ binding. Because changes in dynamic behavior are often indicative of allostery, we conducted HDX-MS experiments on P-Rex1 ± soluble Gβγ.

The dynamic behavior observed in P-Rex1 alone is consistent with our cryo-EM structure and with previous crystallographic data. For example, central β sheets and helices within the IP4P and DH domains exhibit low exchange rates (fig. S9 and data file S1). Conversely, higher exchange rates are observed for the β3/β4 loop of the PH domain and in the helical linker between the DH and PH domains. Linkers joining the PH to the DEP1 domain and DEP1 to the DEP2 domain also exhibit relatively high dynamics. HDX-MS reveals that the αA-αB loop and the 285-residue β5-αC loop contain islands of stable structure around residues 1070 to 1100, 1210 to 1240, and 1290 to 1310 (data file S1) that are predicted to be helical and likely correspond to the low-resolution cryo-EM density that we observe extending from the IP4P domain (fig. S4). Similar extended helical loops are also observed in the crystal structure of SidF (Fig. 3A). This low-resolution density projects near other density that we attribute to the N-terminal DH/PH-DEP1 domains and could make contacts with or at least influence the DH/PH RhoGEF module.

**Fig. 5:**
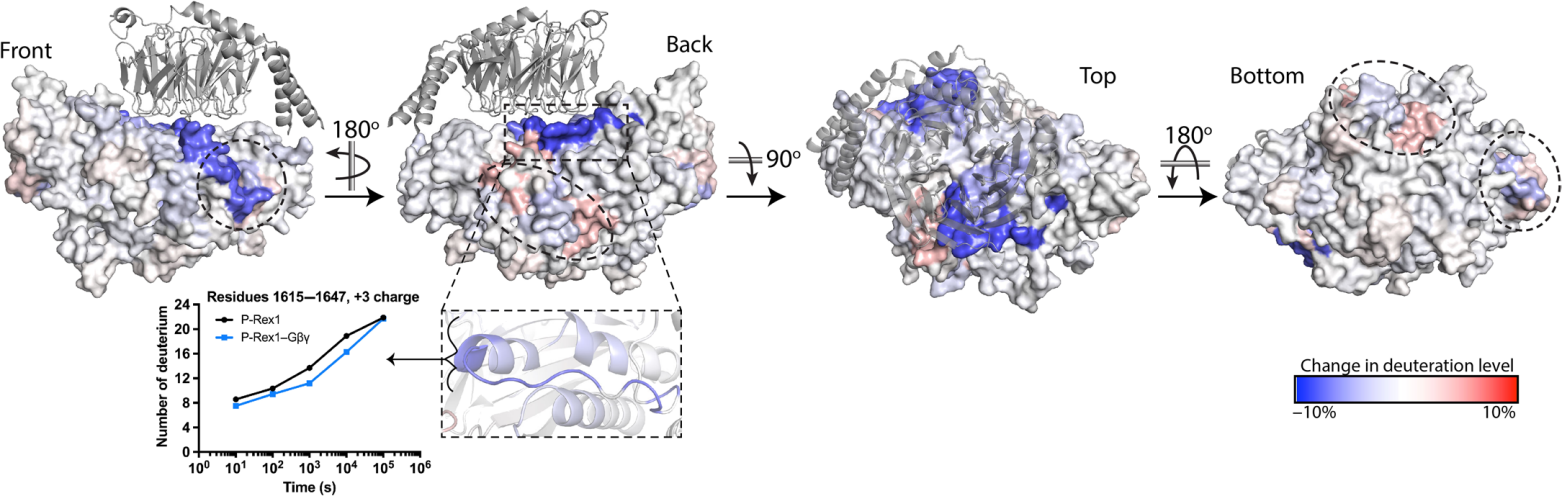
HDX-MS suggests allosteric changes in P-Rex1 upon Gβγ binding. Differences in HDX upon complex formation with Gβγ (at 1000 s) were plotted onto the cryo-EM structure of the P-Rex1 Gβγ-binding module. Red regions, more dynamic behavior upon Gβγ binding; blue regions, less dynamic behavior upon Gβγ binding. Graph shows a comparison of the exchange over time for the indicated structural features. Changes occur distal from the Gβγ-binding site (dashed ovals), suggesting that binding may cause allosteric changes in P-Rex1. These experiments were performed twice, and the data shown represent the average of two experiments. See also data files S1 and S2.

**Fig. 6:**
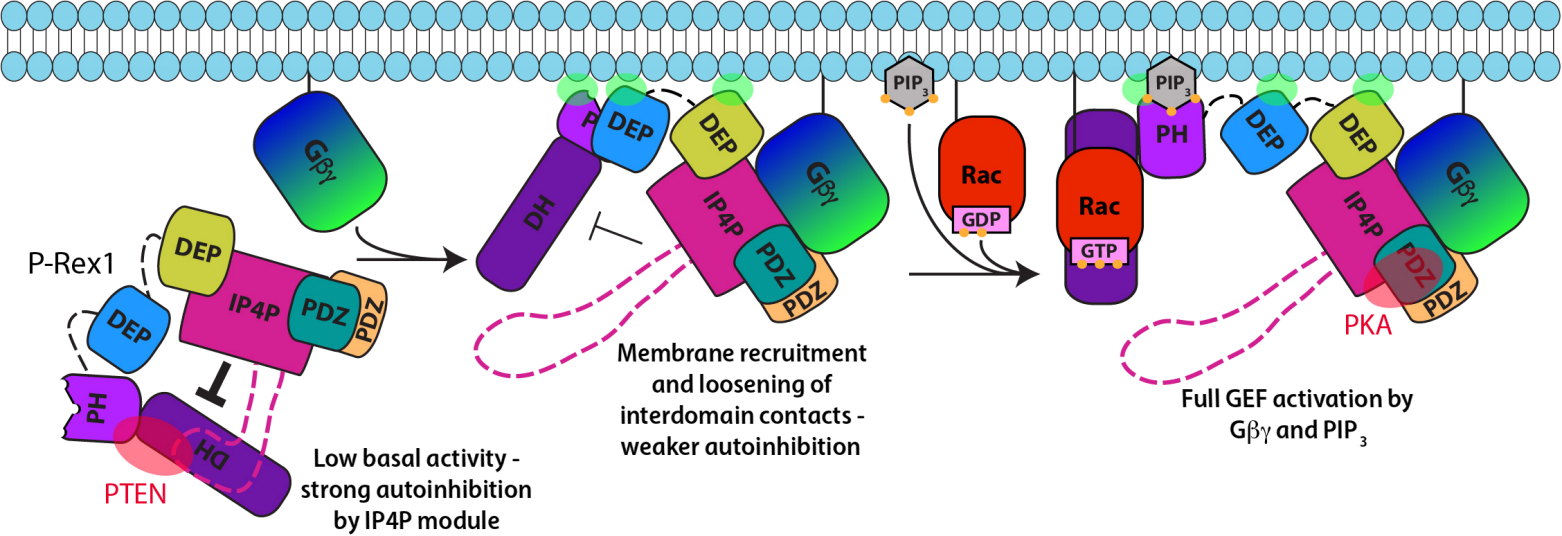
Allosteric activation model for P-Rex1. Our cryo-EM data (fig. S4B) and HDX-MS data (Fig. 5) suggest that the DH/PH/DEP1 domains could interact with the C-terminal Gβγ-binding module, perhaps through the unanticipated domain found within the loops of the IP4P domain (pink dashed line). This low-activity, autoinhibited form is predicted to have weak affinity for the plasma membrane. Gβγ localizes P-Rex1 to the cell membrane and allosterically loosens the autoinhibitory interdomain contacts. Binding to PIP_3_ results in complete activation and provides full substrate access to the RhoGEF active site through an undetermined mechanism. There are multiple points of contact of this complex with the cell membrane, through either lipid modifications (solid black lines) or basic patches on P-Rex1 domains (green transparent ovals). The quaternary arrangement of domains in P-Rex1 is thought to be important for scaffolding interactions with other signaling proteins such as PTEN (specific for P-Rex2) and PKA (red transparent ovals), although these proteins may, in fact, bind P-Rex either at the cell membrane or in the cytoplasm. Dashed lines indicate domains (pink) or flexible linker regions (black) that have not been observed in this or previous structures.

In the presence of Gβγ, the C terminus of P-Rex1 exhibits the most profound decrease in dynamics, exchanging, on average, 30% more slowly at 1000 s (Fig. 5 and data files S1 and S2). Within this interface, the most stabilized peptide segments are those that directly interact with Gβ Trp^99^, consistent with this region of Gβγ being the most important for P-Rex1 activation (Fig. 4D). Likewise, peptides including Gβ Trp^99^ are the most stabilized in Gβγ after incubation with P-Rex1 (data file S1). HDX-MS also provided evidence for long-range allosteric changes within P-Rex1, as regions of PDZ1 and the IP4P domain remote from the Gβγ-binding site (~20 to 40 Å away) exhibited changes in dynamics upon binding (Fig. 5, dashed ovals), consistent with the binding of Gβγ to P-Rex1 causing long-range conformational changes that may lead to release of autoinhibition of the DH domain (Fig. 6). These dynamic changes could partially underlie the increase in P-Rex1 activity observed with Gβγ in the presence of PIP_3_, although membrane localization is still essential (Fig. 4D).

## DISCUSSION

Here, we show that Gβγ binds to an extensive surface on P-Rex1 composed of the PDZ1, PDZ2, and IP4P domains and that activation of P-Rex1 by Gβγ is dependent on its membrane localization and involves allosteric changes. Our structural data will allow the generation of reliable homology models for a new subfamily of mammalian phosphatase domains that includes P-Rex2, INPP4A, and INPP4B. Our data also provide the framework of a signaling scaffold that can integrate signals originating from multiple cell surface receptors and, in response, stimulate cell migration and modulate other pathways that play key roles in normal physiology as well as cancer. Our results also advance our understanding of cancer-associated mutations in P-Rex (Fig. 2) and suggest how other signaling proteins such as PKA and PTEN might interact with P-Rex at the cell membrane (Fig. 6).

Previous discrepancies in the location of the Gβγ-binding site are likely due, in part, to the format of the experiments investigating this question, as these involved studying either one independent domain or constructs containing deletions of one or more domains. Our data show that the overall domain structure of P-Rex1 is complex, and removing a single domain has the potential to cause far-reaching and unexpected consequences in its structural organization, perturbing normal interactions. All our data indicate that the Gβγ-binding site formed by the C-terminal domains of P-Rex1 constitutes the most important site for activation of full-length P-Rex1 by Gβγ under our assay conditions. We do not observe any obvious alternative Gβγ interactions in our cryo-EM data or convincing evidence in our HDX-MS data that an additional binding site for Gβγ can be found on the DH domain (*12*, *14*). However, if Gβγ binds to helical regions of the DH domain, changes in backbone dynamics may not be pronounced. Thus, in light of previous reports, the possibility cannot be ruled out.

The data support the idea that PIP_3_ activates P-Rex1 not through membrane localization to the substrate but through a conformational change induced upon binding (*9*). We and others have furthermore shown that PIP_3_ is required to observe further P-Rex1 activation by Gβγ [Fig. 4D and (*21*)]. Other previous studies have shown that each can independently activate P-Rex1 to some degree (*8*). In experiments where Gβγ was unable to activate P-Rex1 on its own, a soluble GTPase substrate was used, whereas in those that did see Gβγ-mediated activation on its own, a WT substrate was used. We believe that the difference in prenylation of the substrate underlies this discrepancy. With the lipid-tethered substrate, ~10-fold activation was seen with either PIP_3_ or Gβγ, and ~50-fold synergistic activation was seen with both (*8*). In contrast, with soluble GTPase, twofold activation is seen with PIP_3_, and four- to fivefold activation is seen with PIP_3_ plus Gβγ [Fig. 4D and (*21*)]. Thus, colocalization at the membrane, mediated by the WT substrate, would seem to account for a 5- to 10-fold higher activity. Soluble Gβγ (fig. S8D) does not activate under any condition in our assays using a soluble substrate. The PIP_3_-dependent activation by Gβγ in our assays using a soluble GTPase substrate is, however, strong evidence for synergistic allosteric change in P-Rex1 mediated by both activators. Dynamic changes remote from the Gβγ-binding site in our HDX-MS data are consistent with this conclusion.

The P-Rex1–Gβγ complex contains multiple membrane interaction motifs, which helps in modeling its orientation at the cell membrane (Fig. 6). For example, the canonical membrane-binding β1/β2 hairpin loop of the P-Rex1 DEP2 domain is positioned in this complex such that it could readily engage the same lipid bilayer (Fig. 6, green oval) as the prenylated C terminus of Gγ. In this orientation, protein-protein interaction sites within P-Rex1 are positioned to accommodate kinases and phosphatases such as PKA and PTEN, respectively. Type I PKA interacts with the P-Rex1 PDZ domains (Fig. 6, red oval) and phosphorylates the DEP1 domain to promote autoinhibitory interactions (*22*). Phosphorylation of P-Rex1 is known to be important for its regulation, and predicted sites are located throughout the length of the protein (*6*, *23*, *24*). As would be expected, these sites are located in flexible loops (Fig. 2), where they can be readily accessed by protein kinases.

The P-Rex1–Gβγ structure can also be used to clarify the oncogenic roles of P-Rex1 and its close homolog P-Rex2, a related RhoGEF that is highly mutated in cancer (*3*–*5*, *25*–*27*). For example, missense mutations predicted to have the most effect on structure are located near the Gβγ interface, such as P-Rex2 K634E (pancreatic cancer) and A1571E (colorectal cancer), suggesting that they would either interfere with activation by Gβγ or, alternatively, render constitutive activity (Fig. 2). In addition, the PH domain of P-Rex2 interacts with the phosphatase and C2 domains of PTEN (Fig. 6, red oval), a canonical tumor suppressor, while the IP4P domain interacts with the PTEN C terminus (*26*, *28*). This interaction inhibits the activity of both proteins. P-Rex2 mutations V432M, G844D, and P948S are known to allow it to evade inhibition by PTEN in a breast cancer cell invasion assay (*29*). These residues are conserved in P-Rex1 as Val^464^ (solvent-exposed residue in DEP1 domain), Gly^878^ (beginning of αA), and Pro^982^ (end of β4) (Fig. 2). These sites do not cluster closely in the structure, and only Val^464^ is in a region that changes dynamics in the presence of Gβγ. This suggests that perturbation of these positions alters the conformation of P-Rex2 in a manner that globally affects either its ability to be regulated by PTEN or its ability to undergo allosteric change. To better understand the allostery that underlies P-Rex function, future studies will need to examine the structure and dynamics of P-Rex1 when it is directly engaged with membranes via PIP_3_ and Gβγ, as well as in the presence and absence of a substrate GTPase. Given the high quality of structural information provided in this study by cryo-EM, it seems also feasible to obtain structures of the P-Rex1–Gβγ assembly in complex with other scaffolding partners.

## MATERIALS AND METHODS

### Experimental design

The primary objective of this study was to determine the structure of P-Rex1 bound to Gβγ using cryo-EM. We further sought to validate this structure and investigate its dynamics using HDX-MS studies. We also identified the surface on Gβγ that is important for Gβγ-mediated activation of P-Rex1 on liposomes in the presence of PIP_3_.

### Cloning

Human P-Rex1 complementary DNA (cDNA) was a gift from J. Garrison (University of Virginia). The sequence corresponding to residues 38 to 1659 of P-Rex1 was cloned into a pRK5 mammalian expression vector such that the protein expressed would have a Tobacco Etch Virus protease–cleavable, N-terminal glutathione *S*-transferase (GST) fusion and a C-terminal noncleavable 10× tandem histidine tag. Rac1 and Cdc42 expression constructs were described previously (*30*). Site-directed mutations were created using QuikChange (Qiagen) and confirmed by DNA sequencing.

### Protein production and purification

Soluble bovine Gβ_1_γ_2_ was used for cryo-EM experiments and produced as previously described (*31*). Briefly, cells expressing soluble Gβγ were thawed and sonicated using a handheld sonicator (40× 1-s pulses at 9 W) in a buffer of 20 mM HEPES (pH 8), 100 mM NaCl, 0.001 mM leupeptin, 1 mM lima bean trypsin inhibitor, 0.1 mM phenylmethylsulfonyl fluoride (PMSF), and 2 mM dithiothreitol (DTT). Cell lysate was ultracentrifuged in a Ti45 rotor at 40,000 rpm for 45 min at 4°C, and the supernatant was applied to histidine affinity (Ni-NTA) resin equilibrated with 20 mM HEPES (pH 8), 100 mM NaCl, 1 mM MgCl_2_, and 2 mM DTT and then incubated at 4°C while rocking for 1 hour. The supernatant was flowed through the resin, which was then washed extensively with buffer supplemented to 300 mM NaCl and with 10 mM imidazole (pH 8). Gβγ was eluted with buffer supplemented with 200 mM imidazole, and fractions containing Gβγ were diluted threefold with 20 mM HEPES (pH 8), 1 mM MgCl_2_, and 2 mM DTT, then passed through a 0.2-μm filter, and applied to a Mono Q column. Protein was eluted with an NaCl gradient to 500 mM, and fractions containing Gβγ were pooled and concentrated to about 9 mg/ml. Gβγ was then processed over two tandem Superdex 75 10/300 GL columns, pooled, and concentrated to 7.8 mg/ml before flash freezing in liquid nitrogen.

Geranylgeranylated human Gβ_1_γ_2_ (WT and variant) proteins were used for GEF assays and expressed using a double promoter system as described previously (*32*). Gβγ proteins were purified from membrane fractions with Ni-NTA resin as described previously (*33*). The fractions containing Gβγ were pooled and applied to a Superdex 200 column in a buffer containing 20 mM HEPES (pH 8), 100 mM NaCl, 1 mM DTT, and 10 mM CHAPS. Fractions containing Gβγ were concentrated and flash-frozen in liquid nitrogen before storage at −80°C.

Rac1 and Cdc42 were produced and purified as previously described (*9*). Before conjugating to Affi-Gel 10 resin (see below), Rac1 was processed through Superdex 200 resin packed in an XK 16/100 column in a buffer of 50 mM HEPES (pH 7.5), 100 mM NaCl, 1 mM MgCl_2_, and 2 mM DTT.

Recombinant P-Rex1 was expressed in FreeStyle 293-F cells (Thermo Fisher Scientific) using polyethylenimine for transient transfection, and cells were harvested 48 hours later by centrifugation at 1000 relative centrifugal force for 15 min and then frozen in liquid nitrogen. Cells were thawed and lysed in Cell Lytic M (Sigma) supplemented with 200 mM NaCl, 0.1 mM EDTA, 0.001 mM leupeptin, 1 mM lima bean trypsin inhibitor, and 0.1 mM PMSF plus SIGMAFAST Protease Inhibitor Cocktail Tablets (Sigma) at 4°C with rocking for 15 min. Cell lysate was then ultracentrifuged in a Ti45 rotor at 40,000 rpm for 45 min at 4°C, and the supernatant was applied to glutathione agarose resin (Gold Biotechnology Inc.) equilibrated with 20 mM HEPES (pH 8), 200 mM NaCl, 1 mM EDTA, and 2 mM DTT and incubated at 4°C with rocking for 1.5 hours. Supernatant was passed over the resin, which was then washed extensively with equilibration buffer. GST-tagged P-Rex1 was eluted with a buffer of 100 mM HEPES (pH 8), 300 mM NaCl, 2 mM DTT, 1 mM EDTA, and 30 mM reduced glutathione. Elution samples were simultaneously digested with TEV protease to remove the GST tag and dialyzed overnight at 4°C into a buffer of 20 mM HEPES (pH 8), 100 mM NaCl, 1 mM EDTA, and 2 mM DTT. P-Rex1 was then either used for HDX-MS and nucleotide exchange experiments or further purified over a Mono Q 5/50 GL anion exchange column (GE Healthcare Life Sciences), eluted with an NaCl gradient to 300 mM, and concentrated, with final P-Rex1 sample purity of >85%. For cryo-EM experiments, a substrate affinity column was created to obtain >98% pure, high-quality P-Rex1 by conjugating Rac1 to Affi-Gel 10 resin (Bio-Rad) as per the manufacturer’s protocol. The dialyzed P-Rex1 sample was applied to this Rac1 column and incubated at 4°C with rocking for 15 min, and then EDTA was added to a 10 mM final concentration followed by incubation for a further 1.5 hours. Unbound protein was washed off, and the resin was washed with 2.5 column volumes of wash buffer [20 mM HEPES (pH 8), 100 mM NaCl, 1 mM EDTA, and 2 mM DTT], followed by 2.5 column volumes of wash buffer without EDTA. P-Rex1 was eluted from the column with a buffer of 20 mM HEPES (pH 8), 200 mM NaCl, 5% glycerol, 2 mM DTT, 3 mM MgCl_2_, and 10 μM guanosine diphosphate (GDP) in several tandem 1-ml fractions. EDTA was immediately added to each to a final concentration of 4 mM, and fractions containing P-Rex1 were pooled and concentrated to 0.9 mg/ml (fig. S1A).

### Cryo-EM grid preparation and data acquisition

For cryo-EM sample preparation, P-Rex1 was mixed with soluble Gβγ to final concentrations of 3 and 6 μM, respectively, and *n*-dodecyl-β-d-maltoside (DDM) was added to a final concentration of 0.08 mM. A sample of 4 μl of this mixture was applied to either a glow-discharged Quantifoil (1.2/1.3) 300-mesh grid for untilted data collection or UltraAuFoil R (1.2/1.3) 300-mesh gold grid (Electron Microscopy Sciences) for tilted data collection. The grids were then blotted with filter paper and plunge-frozen into ethane cooled with liquid nitrogen using a Vitrobot Mark IV (Thermo Fisher Scientific) set to 4°C, 100% humidity, 4-s blot, and a force of 20. Micrographs were collected using Leginon (*34*) on a Titan Krios transmission electron microscope (Thermo Fisher Scientific) operating at 300 keV using a Gatan K2 Summit direct electron detector (Gatan Inc.) in counting mode (1 Å/pixel) at a nominal magnification of 29,000×.

### Image processing

Initial attempts at determining this structure revealed a preferred orientation of the complex on cryo-EM grids, limiting its resolution to ~6 Å. We overcame this by collecting and merging together 0° and 30° tilted cryo-EM data, resulting in 905,464 particles that were used to generate a 3.2 Å map. Untilted and tilted datasets were preprocessed separately (table S1). For each dataset, micrograph assessment, particle picking, and contrast transfer function estimation were performed using Warp (*35*), resulting in 600,588 particles (0°) and 304,876 particles (30°). These particles were merged together and underwent 2D image classification into 75 classes using cryoSPARC v0.65 (fig. S1) (*36*). After removing particles within bad classes, the remaining 611,231 particles were used for ab initio reconstruction into three classes using cryoSPARC (fig. S2). From the highest resolution model, 205,599 particles were selected for homogeneous refinement in cryoSPARC to obtain a 3.2 Å structure of the P-Rex1–Gβγ complex (fig. S3). Particles in this model were further classified using RELION (*37*) into 10 classes. One of these classes displayed density for both the P-Rex1 N terminus and loops extending from the IP4P domain and was selected for 3D refinement in RELION, reaching a resolution of 9.6 Å (fig. S4).

### Model building and refinement

To begin modeling into our map of the P-Rex1–Gβγ complex, the program phenix.dock_in_map (*38*) was used to place atomic structures for Gβγ (PDB: 3V5W), residues 622 to 706 of PDZ1 (PDB: 3QIK), and threaded models of PDZ2 (residues 707 to 788) created by i-Tasser and DEP2 (residues 508 to 599) created by Swiss-Model along with the sequences for Gβγ and P-Rex1 residues 497 to 1659. The program phenix.map_to_model (*39*) was used along with the same sequence input and the partial model generated in phenix.dock_in_map to generate Cα backbone traces in unmodeled regions of the map with clear secondary structure. This method generated a number of helices and β-strands within the IP4P domain. The Cα coordinates for these were then submitted to the Dali webserver to search for homologous structures in the PDB, which identified *Legionella* phosphoinositide phosphatases SidF (PDB: 4FYG) (*16*) and SidP (PDB: 4JZA) (*15*) to be among the highest hits. These enzymes share the C*X*_5_R P loop catalytic motif located near the C terminus of P-Rex1 (residues 1583 to 1589). Using this motif as an anchoring point, SidF and SidP were aligned with the core of the IP4P domain. Building outward from this region, the homologs were used to help identify the connectivity as necessitated by the disordered or poorly ordered loops linking many of the secondary structure elements in the IP4P domain. Model building was performed in Coot, and structure refinement and model validation were performed in PHENIX using phenix.real_space_refine (fig. S3, D to F, and table S1) (*40*). To make figures showing map density, phenix.map_box was used to restrict the map shown to specific stretches of residues. The PyMOL Molecular Graphics System (version 2.1, Schrödinger, LLC) was used to render images showing these and other structure renditions. To calculate surface electrostatics, PDB files were run through the PDB2PQR server using a PARSE forcefield and PROPKA to perform p*K*_a_ (where *K*_a_ is the acid dissociation constant) calculations at pH 8 and create an APBS input file, and then APBS was run to generate a file for electrostatic surface visualization using the same server. In figures, electrostatic surface potentials were colored on a scale of −20 (red) to 20 (blue) *k*_B_*T*/*e*_c_. UCSF (University of California, San Francisco) Chimera was used to create images showing cryo-EM envelopes. Sequence alignment shown in Fig. 2 was produced using Clustal Omega, and buried surface area was calculated using PISA.

### Phosphatase activity assay

P-Rex1 phosphatase activity was measured using a malachite green phosphatase assay kit (Echelon Biosciences), PIP substrates (Echelon Biosciences), and phosphorylated peptides derived from substrates for protein tyrosine phosphatase (PTP) and protein phosphatase 2 (PP2; Enzo Life Sciences). Assays were performed in a 384-well, clear plate in 20 mM HEPES (pH 8), 200 mM NaCl, and 1 mM tris(2-carboxyethyl)phosphine with a total reaction volume of 10 μl. GST-tagged P-Rex1 WT and variant C1583A were purified using glutathione agarose resin and then used in the assay without removing the tag. P-Rex1 was tested at 1 μM, Gβγ at 2 μM, lipid substrates at 200 μM, and peptide substrates at 500 μM. After combining reagents, the plate was sealed with clear sealing film and incubated at room temperature for 3 hours. Forty microliters of malachite green was then added to each reaction and incubated for 15 min before reading absorbance at 620 nm. A phosphate standard was analyzed with each set of reactions and used to calculate the amount of liberated phosphate in each reaction.

### GTPase exchange activation assay

P-Rex1 GEF activity was measured via loss of fluorescence upon dissociation of fluorescently labeled *N*-methyl-anthraniloyl-GDP (mant-GDP; Jena Bioscience) from unprenylated Cdc42. We chose to use soluble (unprenylated) Cdc42 so that we could detect allosteric effects of P-Rex1 activation by Gβγ and PIP_3_ as opposed to effects due to colocalization with Cdc42 at the liposome. Cdc42 was first incubated with twofold molar excess of mant-GDP in 20 mM HEPES (pH 8), 100 mM NaCl, 4 mM EDTA, and 1 mM DTT for 2 hours on ice. To stabilize the loaded mant-GDP, MgCl_2_ was added to a final concentration of 5 mM, and the sample was incubated for 1 hour on ice. Subsequently, the mant-GDP–loaded Cdc42 was exchanged into reaction buffer [20 mM HEPES (pH 8), 100 mM NaCl, 5 mM MgCl_2_, and 1 mM DTT] via a gel filtration column (Bio-Gel P-30; Bio-Rad) preequilibrated with reaction buffer to remove excess nucleotides. Liposomes in the nucleotide exchange assay were composed of 200 μM each of phosphatidylserine (PS) (16:0/18:1; Avanti Polar Lipids Inc.), phosphatidylcholine (PC) (16:0/18:1), and varying concentrations of PIP_3_ (Cayman Chemical Company). The liposomes were prepared as 10× stocks by combining liquid chloroform stocks together and then drying the solvent under nitrogen gas. The lipid film layer was further desiccated for 2 hours before resuspension in 20 mM HEPES (pH 8) and 100 mM NaCl. The lipid solution was mixed and sonicated in a water bath until it became clear. The liposomes were either used fresh or stored at 4°C and used within 3 to 4 days of generation. P-Rex1 (100 nM) and Gβγ proteins at various concentrations were incubated with the liposomes in 20 mM HEPES (pH 8), 100 mM NaCl, 0.5 mM MgCl_2_, 100 μM guanosine triphosphate (GTP), and 1 mM DTT for 20 min at room temperature, and the reaction was initiated by addition of 2 μM mant-GDP–loaded Cdc42. For the experiments with Gβγ variants, 0.5 μM PIP_3_ and 250 nM Gβγ were used. For Gβγ dose-response curves, 0.5 μM PIP_3_ was included in the liposomes. The fluorescence (λ_ex_ = 360, λ_ex_ = 440 nm) was measured at 25°C for 1 hour. The rate was calculated by using the one-phase exponential decay model in Prism.

### Hydrogen-deuterium exchange mass spectrometry

To generate the best proteolytic peptide coverage map for performing HDX-MS experiments, the quench condition was first optimized for P-Rex1. P-Rex1 [3 μl of 1.3 mg/ml in 10 mM tris-HCl (pH 7.2), 150 mM NaCl, and 2 mM DTT] was mixed with 9 μl of H_2_O buffer at 0°C, to which was added 18 μl of ice-cold quench buffers containing 0.1 M glycine (pH 2.4), 16.6% glycerol, and various concentrations of guanidinium hydrochloride (GuHCl) (0.08, 0.8, 1.6, and 3.2 M). The quenched samples were then subjected to an immobilized pepsin column (16-μl bed volume) on ice at a flow rate of 20 μl/min for online digestion. Proteolytic products were collected on a trap column for desalting, and liquid chromatography–MS (LC-MS) analyses were performed on an Agilent Poroshell C18 column (EC-C18, 35 mm × 0.3 mm, 2.7 μm) with a linear gradient of acetonitrile of 6.4 to 38.4% over 30 min. Both trap and C18 columns were kept at 0°C. MS analysis was done using an OrbiTrap Elite mass spectrometer (Thermo Fisher Scientific, San Jose, CA), and MS/MS data were searched against a single protein database of P-Rex1 by Proteome Discoverer. The coverage maps of identified peptides were compared with each other, and ultimately, 0.8 M GuHCl quench buffer was selected.

Exchange stock solutions of P-Rex1 and the P-Rex1–Gβγ complex were prepared in a buffer of 8.3 mM tris (pH 7.2) and 150 mM NaCl. The P-Rex1–Gβγ complex was made by mixing P-Rex1 with Gβγ at a 1:1.6 molar ratio. At 0°C, 30 μl of free P-Rex1 or P-Rex1–Gβγ complex was mixed with 90 μl of D_2_O buffer (8.3 mM tris, 150 mM NaCl, pD reading of 7.2) to initiate the HDX reaction. At various times (10, 100, 1000, 10,000 and 100,000 s), 12 μl of exchange reaction solution was mixed with 18 μl of ice-cold quench buffer (0.8 M GuHCl) to quench the reaction and then immediately frozen on dry ice. Nondeuterated and fully deuterated control samples were also prepared for back exchange correction. All frozen samples were thawed at 4°C and subjected to the above system for enzymatic digestion, LC separation, and MS analysis. All the columns were kept at 0°C to minimize back exchange. The extent of deuterium incorporation into peptides was determined using the specialized software HDExaminer (Sierra Analytics, LLC, Modesto, CA), which calculates centroid values of each peptide. Ribbon maps were generated with an in-house Excel macro and MATLAB scripts. P-Rex1 alone and the P-Rex1–Gβγ complex were each analyzed twice by HDX-MS, and the data shown represent the average of these experiments.

### Statistical analysis

All statistical analyses of GEF activity assays were performed using GraphPad Prism 7 software (GraphPad Software Inc., La Jolla, CA) on data from three or more independent experiments. For comparison of P-Rex1 GEF activity in the presence or absence of Gβγ variants (Fig. 4D), error bars are the mean ± 95% confidence interval from three independent experiments, two of which were performed in duplicate. ***P = 0.0002 and ****P < 0.0001 calculated using one-way analysis of variance with Dunnett’s multiple comparisons posttest.

## Supplementary Material

http://advances.sciencemag.org/cgi/content/full/5/10/eaax8855/DC1

Download PDF

Data file S1

Data file S2

Cryo–electron microscopy structure and analysis of the P-Rex1–Gβγ signaling scaffold

## SUPPLEMENTARY MATERIALS

Supplementary material for this article is available at http://advances.sciencemag.org/cgi/content/full/5/10/eaax8855/DC1 Fig. S1. Sample preparation and cryo-EM images of P-Rex1–Gβγ. Fig. S2. Cryo-EM data processing overview. Fig. S3. Validation of cryo-EM reconstruction. Fig. S4. Cryo-EM map of the P-Rex1–Gβγ complex from fig. S2E showing the P-Rex1 N-terminal region and loops extending from the IP4P domain core. Fig. S5. Comparison of the independently crystallized P-Rex1 PDZ1 domain (PDB: 3QIK) with the PDZ1 domain in the context of the P-Rex1 C-terminal core. Fig. S6. Full-length P-Rex1 lacks phosphatase activity. Fig. S7. Detailed view of P-Rex1–Gβγ interaction sites. Fig. S8. Effects of PIP_3_ and Gβγ on guanine nucleotide exchange accelerated by P-Rex1. Fig. S9. HDX-MS of P-Rex1 alone. Table S1. Cryo-EM data collection, refinement, and validation statistics. Data file S1. Ribbon maps representing HDX-MS experiments. Data file S2. Kinetic data for HDX-MS experiments.

## Appendix

View/request a protocol for this paper from *Bio-protocol*.
