# Supplementary material for: Cryo–electron microscopy structure and analysis of the P-Rex1–Gβγ signaling scaffold
Source: Sci Adv. 2019 Oct 16;5(10):eaax8855. doi: 10.1126/sciadv.aax8855 (PMC6795519; doi:10.1126/sciadv.aax8855)
Supplement: http://advances.sciencemag.org/cgi/content/full/5/10/eaax8855/DC1 [file supp_5_10_eaax8855__index.html]

Science Advances | Science AdvancesAAASSearchScience AdvancesMenu

## Supplementary Materials

**The PDF file includes:**

- Fig. S1. Sample preparation and cryo-EM images of P-Rex1–Gβγ.
- Fig. S2. Cryo-EM data processing overview.
- Fig. S3. Validation of cryo-EM reconstruction.
- Fig. S4. Cryo-EM map of the P-Rex1–Gβγ complex from fig. S2E showing the P-Rex1 N-terminal region and loops extending from the IP4P domain core.
- Fig. S5. Comparison of the independently crystallized P-Rex1 PDZ1 domain (PDB: 3QIK) with the PDZ1 domain in the context of the P-Rex1 C-terminal core.
- Fig. S6. Full-length P-Rex1 lacks phosphatase activity.
- Fig. S7. Detailed view of P-Rex1–Gβγ interaction sites.
- Fig. S8. Effects of PIP3 and Gβγ on guanine nucleotide exchange accelerated by P-Rex1.
- Fig. S9. HDX-MS of P-Rex1 alone.
- Table S1. Cryo-EM data collection, refinement, and validation statistics.
- Legends for data files S1 and S2

Download PDF

**Other Supplementary Material for this manuscript includes the following:**

- Data file S1 (.pdf format). Ribbon maps representing HDX-MS experiments.
- Data file S2 (Microsoft Excel format). Kinetic data for HDX-MS experiments.

**Files in this Data Supplement:**

- Adobe PDF - aax8855\_SM.pdf
- Adobe PDF - aax8855\_Data\_file\_S1.pdf
