# Supplementary material for: Cryo–electron microscopy structure and analysis of the P-Rex1–Gβγ signaling scaffold
Source: Sci Adv. 2019 Oct 16;5(10):eaax8855. doi: 10.1126/sciadv.aax8855 (PMC6795519; doi:10.1126/sciadv.aax8855)
Supplement: Download PDF [file aax8855_SM.pdf]

Science Advances
AAAS

advances.sciencemag.org/cgi/content/full/5/10/eaax8855/DC1

Supplementary Materials for

Cryo–electron microscopy structure and analysis of the P-Rex1–Gβγ signaling scaffold

Jennifer N. Cash, Sarah Urata, Sheng Li, Sandeep K. Ravala, Larisa V. Avramova, Michael D. Shost, J. Silvio Gutkind, John J. G. Tesmer*, Michael A. Cianfrocco*

*Corresponding author. Email: jtesmer@purdue.edu (J.J.G.T.); mcianfro@umich.edu (M.A.C.)

Published 16 October 2019, *Sci. Adv.* **5**, eaax8855 (2019)
DOI: 10.1126/sciadv.aax8855

The PDF file includes:

Fig. S1. Sample preparation and cryo-EM images of P-Rex1–Gβγ.
Fig. S2. Cryo-EM data processing overview.
Fig. S3. Validation of cryo-EM reconstruction.
Fig. S4. Cryo-EM map of the P-Rex1–Gβγ complex from fig. S2E showing the P-Rex1 N-terminal region and loops extending from the IP4P domain core.
Fig. S5. Comparison of the independently crystallized P-Rex1 PDZ1 domain (PDB: 3QIK) with the PDZ1 domain in the context of the P-Rex1 C-terminal core.
Fig. S6. Full-length P-Rex1 lacks phosphatase activity.
Fig. S7. Detailed view of P-Rex1–Gβγ interaction sites.
Fig. S8. Effects of PIP_3 and Gβγ on guanine nucleotide exchange accelerated by P-Rex1.
Fig. S9. HDX-MS of P-Rex1 alone.
Table S1. Cryo-EM data collection, refinement, and validation statistics.
Legends for data files S1 and S2

Other Supplementary Material for this manuscript includes the following:

(available at advances.sciencemag.org/cgi/content/full/5/10/eaax8855/DC1)

Data file S1 (.pdf format). Ribbon maps representing HDX-MS experiments.
Data file S2 (Microsoft Excel format). Kinetic data for HDX-MS experiments.

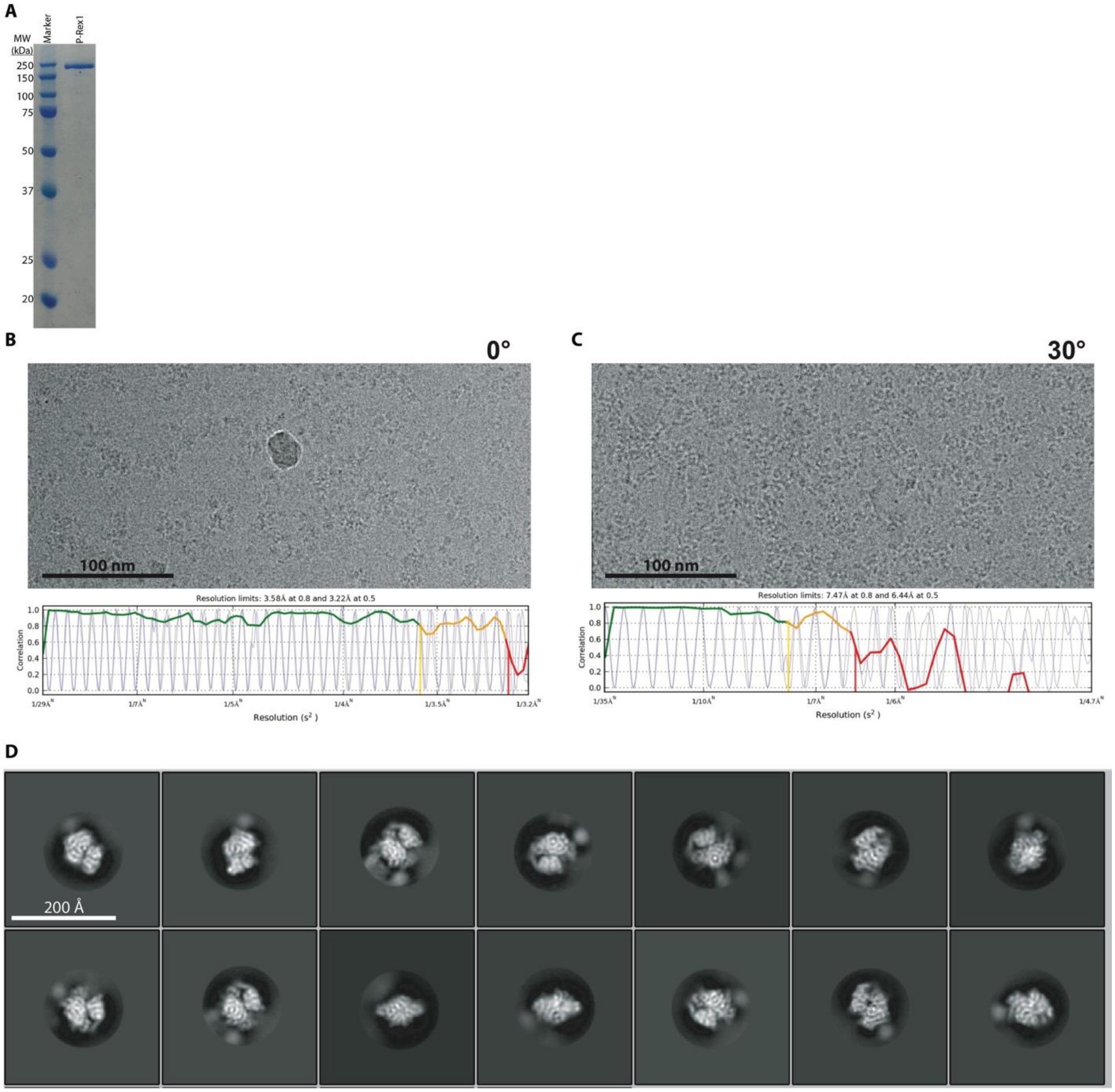

Fig. S1. Sample preparation and cryo-EM images of P-Rex1–Gβγ. (**A**) Coomassie SDS-PAGE showing purified human P-Rex1. (**B**) Representative image of a cryo-EM micrograph from the untilted P-Rex1–Gβγ grid (top). Plot of 1-dimensional contrast transfer function (CTF) using a grey line for micrograph Thon rings and a blue line for estimated CTF using CTFFIND4 (bottom). Green line indicates high confidence fit, dropping below 0.5 confidence at 3.2 Å. (**C**) Representative image of a cryo-EM micrograph for P-Rex1–Gβγ grids tilted at 30° (top). Confidence fit drops below 0.5 at 6.4 Å. (**D**) Representative 2D class averages of P-Rex1 bound to Gβγ from merged tilted and untilted data sets using RELION classification.

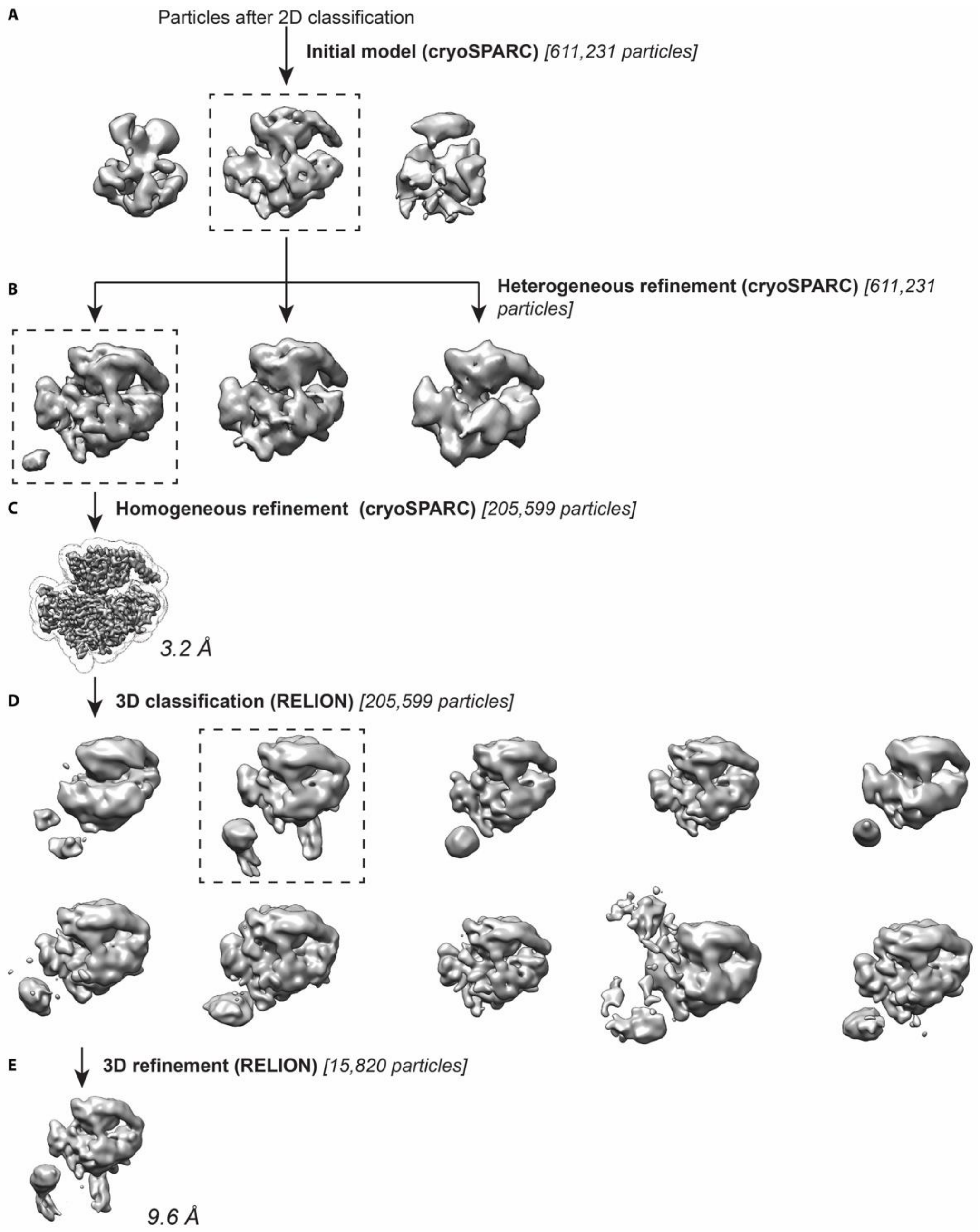

Fig. S2. Cryo-EM data processing overview. (**A**) After 2D classification in cryoSPARC to remove contaminants, initial 3D structures were calculated using *ab initio* model generation in cryoSPARC, asking for three models. Only one structure (dashed box) showed interpretable structural features, and this was selected as a seed for heterogenous refinement into three classes in cryoSPARC (**B**). Following heterogenous refinement, one class had the most homogenous and high-resolution features (dashed box), and this was then used for homogenous refinement in cryoSPARC (**C**). Note that a mask was used during refinement to exclude flexible domains outside of the stable P-Rex1 Gβγ-binding module–Gβγ complex. (**D**) Using the Euler angles from cryoSPARC, the same particles used for 3D homogenous refinement were then subjected to 3D classification into 10 classes in RELION using local angular searches. One class showed additional structured densities and was subjected to 3D refinement in RELION (**E**).

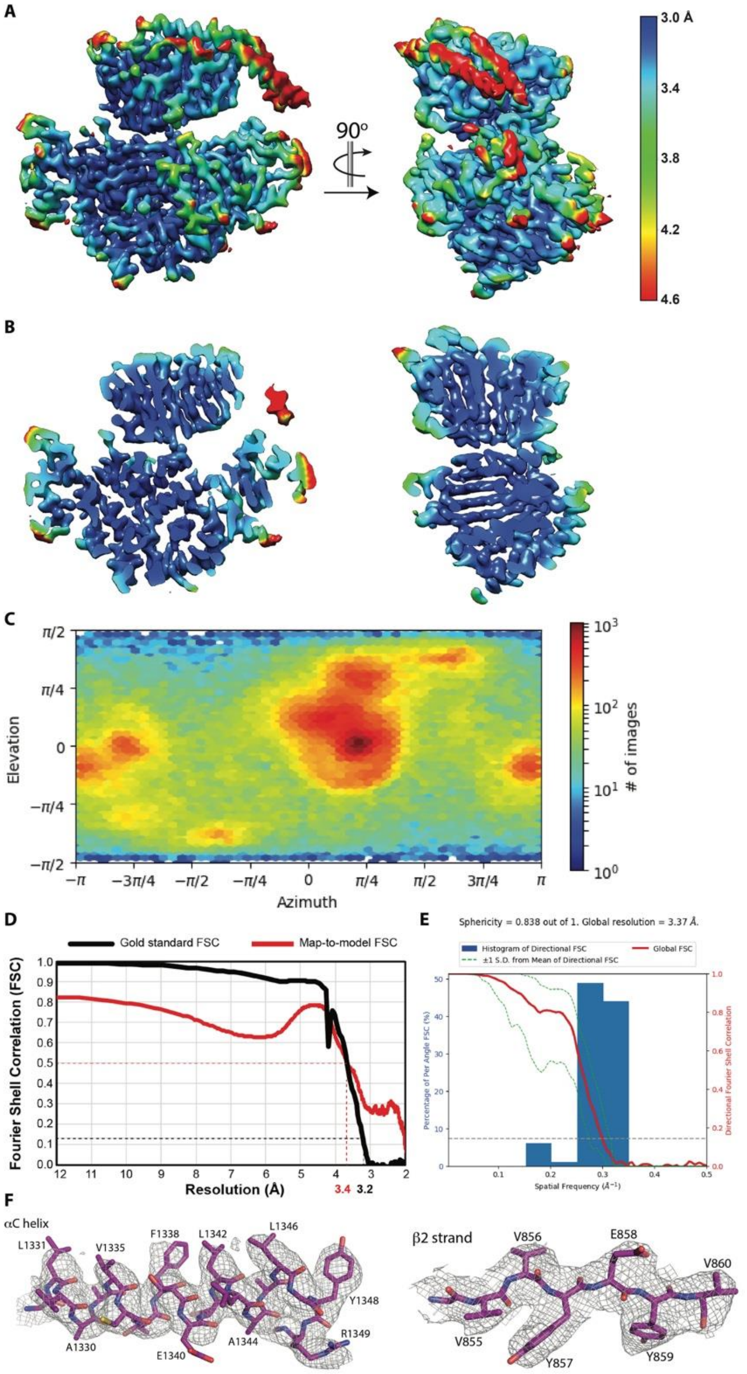

Fig. S3. Validation of cryo-EM reconstruction. (**A**) Cryo-EM structure of P-Rex1–Gβγ colored according to local resolution calculated by cryoSPARC. (**B**) Slices through cryo-EM density in **A**, highlighting quality of map. (**C**) Euler angle distribution of cryo-EM structure highlighting the preferred orientation of the complex on cryo-EM grids and the need for tilted images. (**D**) FSC curves showing the gold standard FSC curve for the final 3D reconstruction in cryoSPARC (black) and a map-to-model FSC calculated between the atomic model and 3D reconstruction using Phenix (red). (**E**) Directional FSC histogram for the final 3D reconstruction. (**F**) Selected cryo-EM densities from the P-Rex1–Gβγ map and fit atomic model.

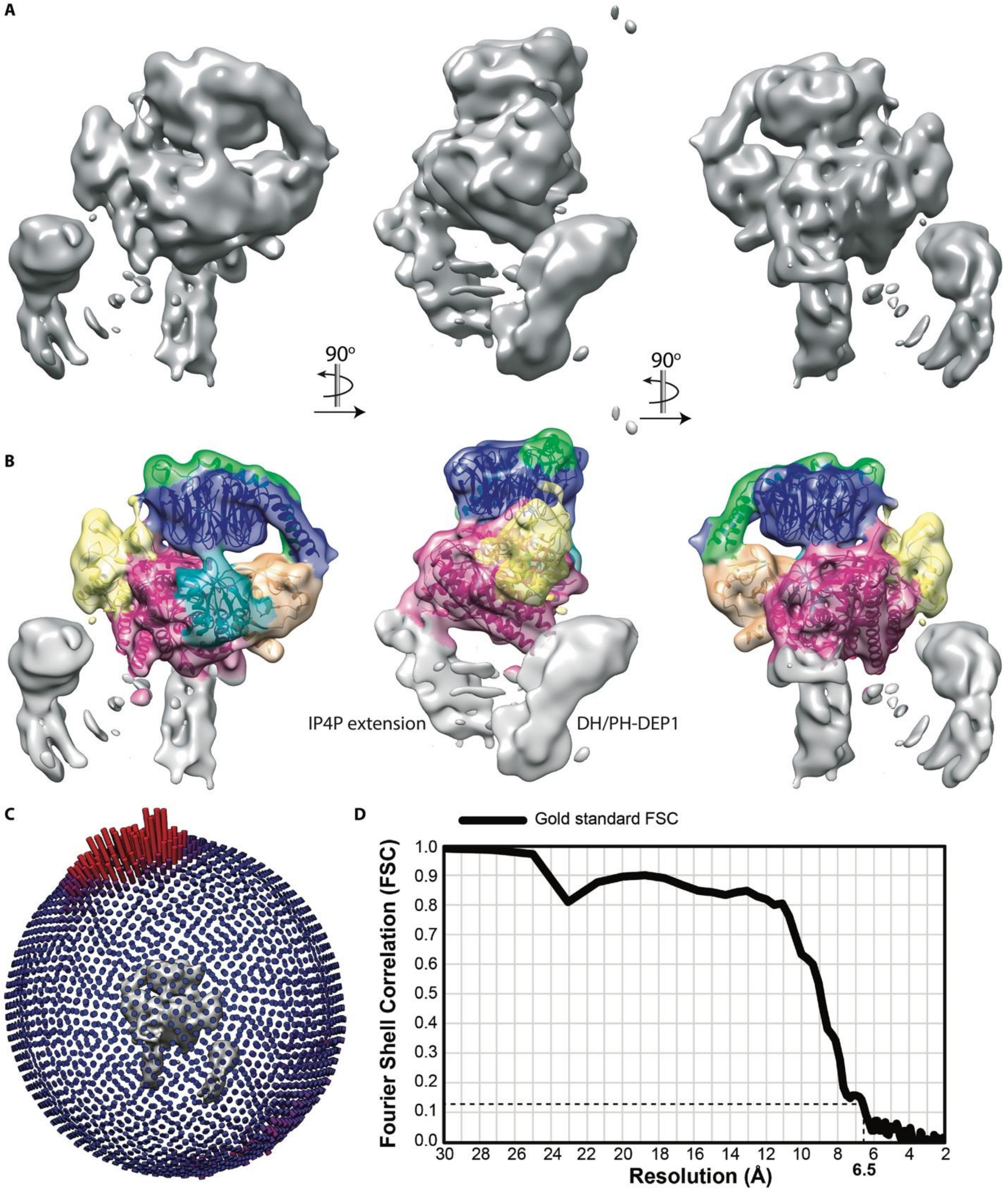

Fig. S4. Cryo-EM map of the P-Rex1–Gβγ complex from fig. S2E showing the P-Rex1 N-terminal region and loops extending from the IP4P domain core. (**A**) Cryo-EM structure filtered at 9 Å. (**B**) Map colored according to the P-Rex1–Gβγ structure as shown in Fig. 1. (**C**) Euler angle distribution. (**D**) Gold standard FSC curve for the final reconstruction.

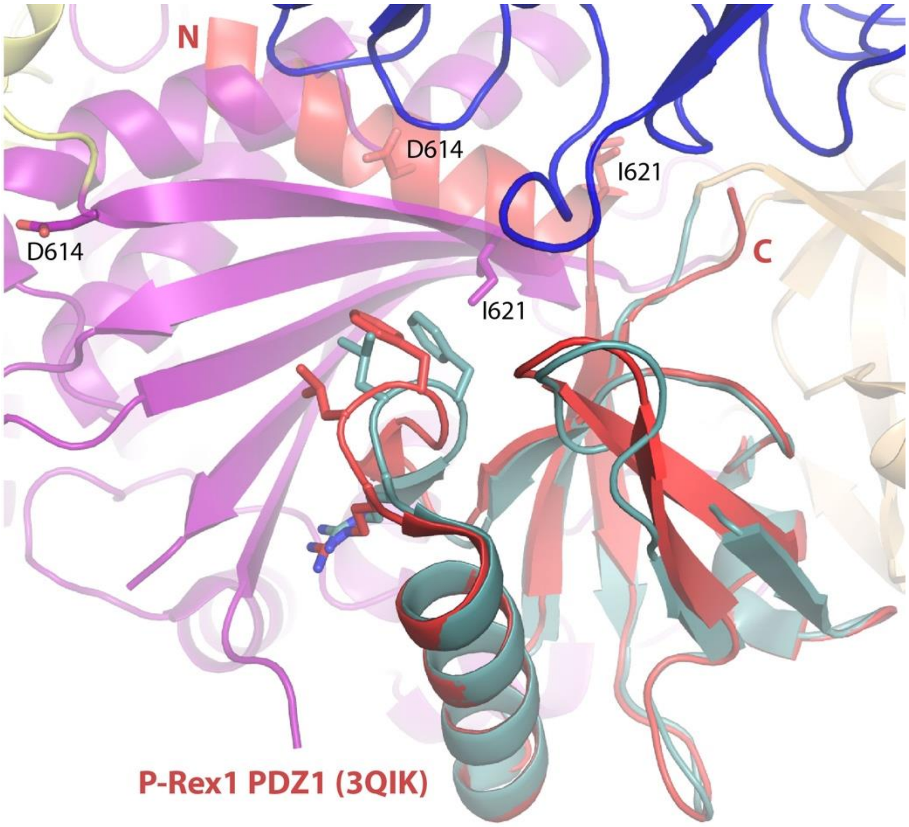

Fig. S5. Comparison of the independently crystallized P-Rex1 PDZ1 domain (PDB: 3QIK) with the PDZ1 domain in the context of the P-Rex1 C-terminal core. Shifts are seen in the loops proximal to Gβγ (blue cartoon), and there is a striking difference in the structure of the β-stand linking the DEP2 and PDZ1 domains (residues 606-623), which instead forms a helix in the crystal structure of the independent PDZ1 domain as well as coiled-coil interactions with symmetry related subunits (compare positions of residues Asp614 and Ile621). Select residues in the PDZ domain are shown to highlight conformational changes that may be a result of Gβγ interaction.

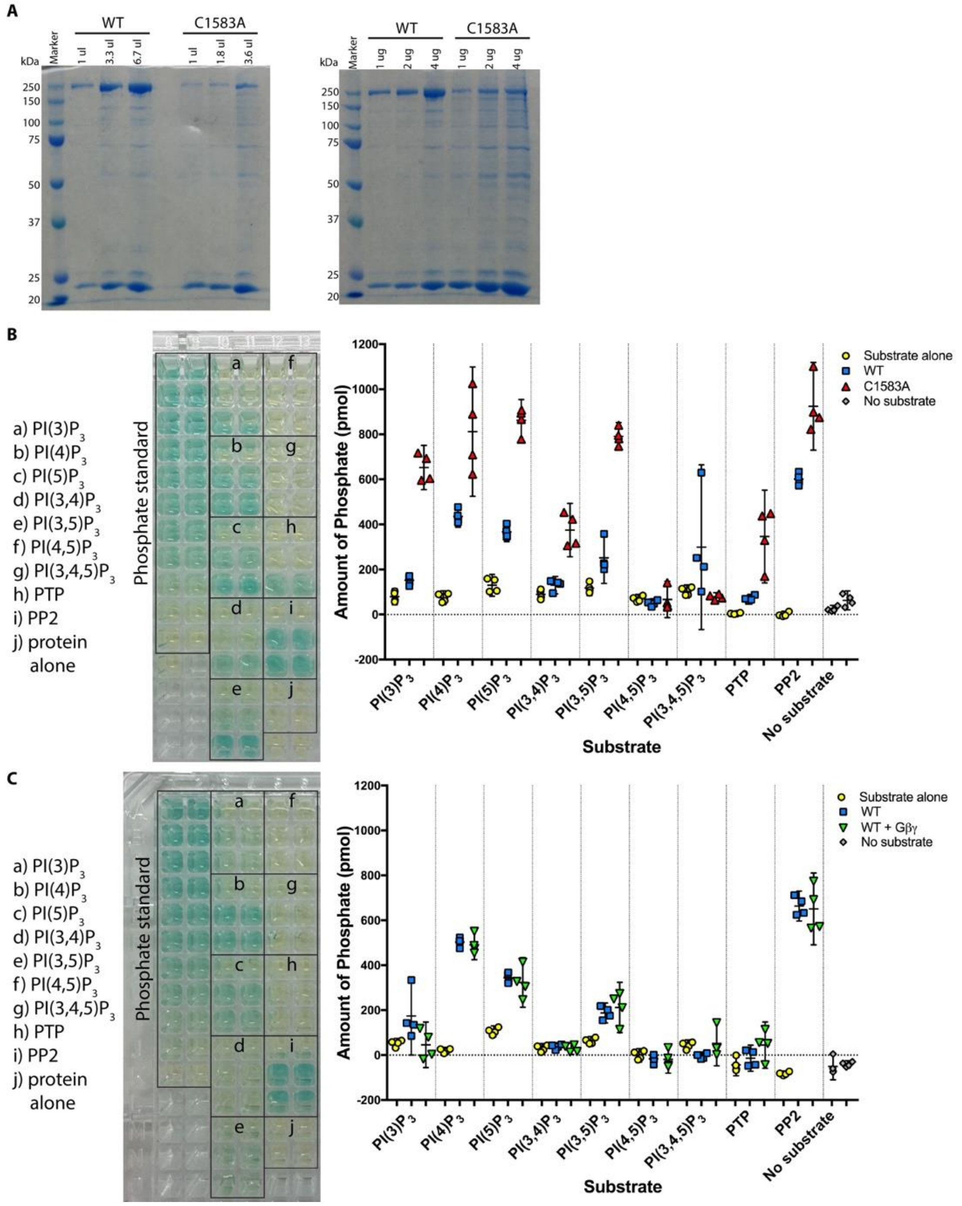

Fig. S6. Full-length P-Rex1 lacks phosphatase activity. (**A**) SDS-PAGE comparing purity of P-Rex1 WT and C1583A used in this assay. The variant expressed poorly, resulting in lower yield and more impurity. The gel on the right was loaded based on estimation of total protein in each sample. (**B**,**C**) WT and C1583A P-Rex1 (**B**) were tested in a malachite green phosphatase assay ± soluble Gβγ (**C**). Left panels show sets of samples for a representative experiment. Within each box of samples, the top row contains substrate alone, middle row is substrate plus P-Rex1 WT, and bottom row is the remaining sample in the set (either C1583A or + Gβγ), with each in duplicate. Each experiment was performed twice in duplicate, and error bars represent 95% confidence intervals. The phosphatase activity seen in samples containing P-Rex1 is most likely attributable to contaminating phosphatases, which are more abundant in the C1583A variant preparation. Although P-Rex1 was used at 1 μM in this assay, phosphatases such as PTEN and SidF are active at concentrations around 10-100 nM. Gβγ had no effect on the phosphatase activity observed.

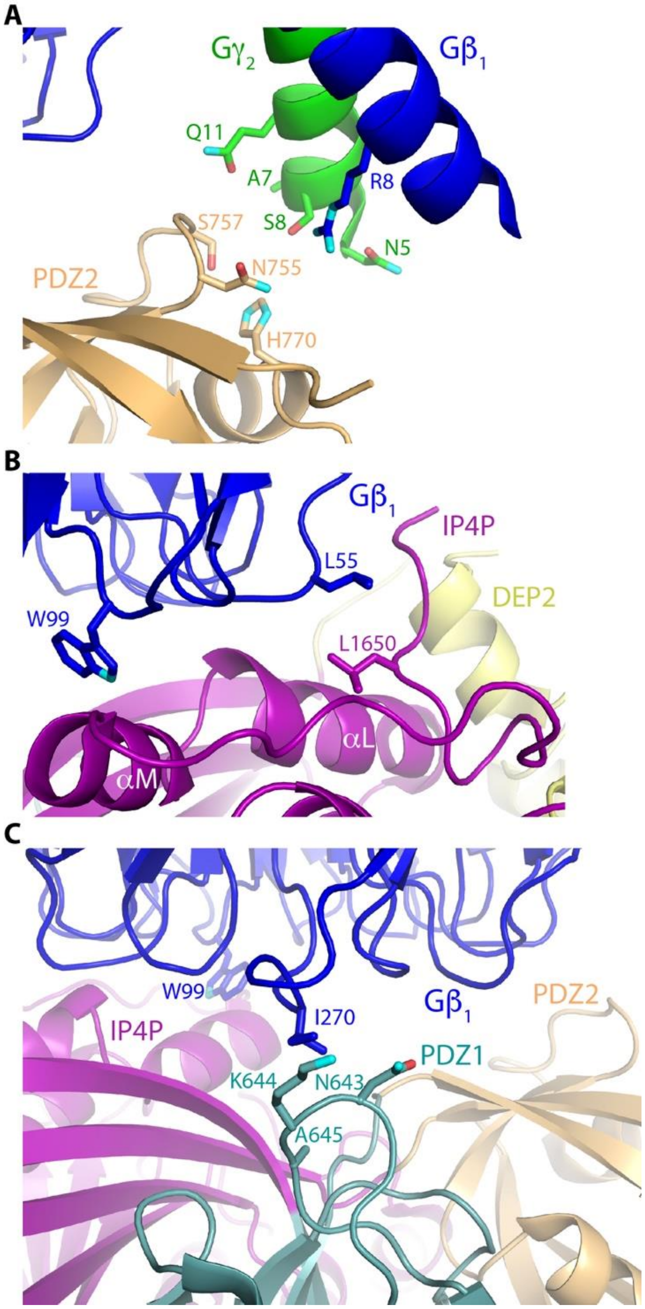

Fig. S7. Detailed view of P-Rex1–Gβγ interaction sites. (**A**) Interactions of the Gβγ N-terminal helices with the P-Rex1 PDZ2 domain, showing residues that have buried surface area upon complex formation. (**B**, **C**) Interactions of Gβ with the P-Rex1 IP4P and PDZ1 domains, respectively. Gβ Trp99 is shown in both panels as a reference.

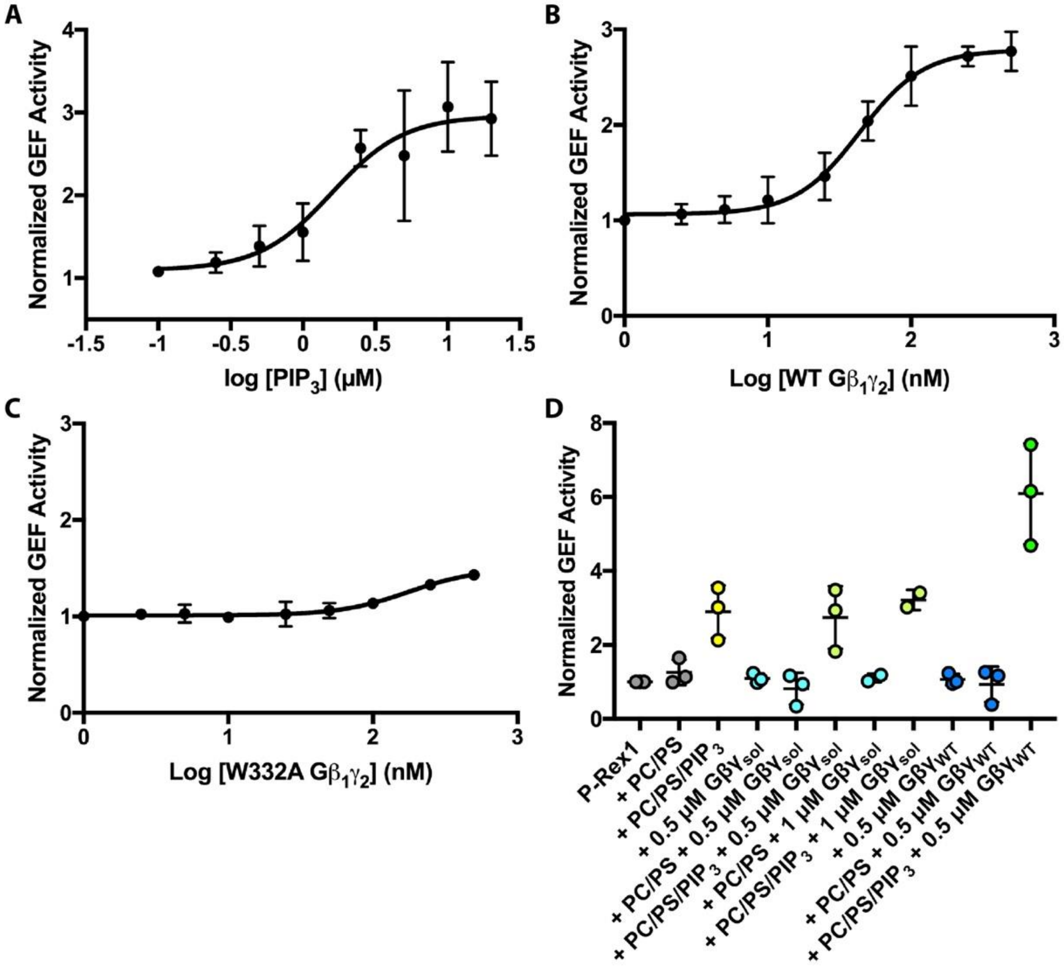

Fig. S8. Effects of PIP$_3$ and Gβγ on guanine nucleotide exchange accelerated by P-Rex1. Liposome-based guanine nucleotide exchange assay with unprenylated Cdc42 demonstrating dependence of P-Rex1 activation on PIP$_3$ and Gβγ. (**A**) Liposomes contained 200 μM each of PC and PS and increasing concentrations of PIP$_3$. Exchange activity was normalized to that in the absence of PIP$_3$. EC$_{50}$= 1.6 μM (95% CI 0.82 to 4.2) from 6 independent experiments. (**B**) Liposomes contained 200 μM each PC and PS and 0.5 μM PIP$_3$ along with increasing concentrations of WT Gβγ. The final concentration of CHAPS in these experiments was 0.05 mM. Exchange activity in each experiment was normalized to that in the absence of Gβγ. EC$_{50}$= 44 nM (95% CI 35 to 55) from 4 independent experiments. (**C**) The Gβ$_{W332A}$γ variant was similarly examined and showed relatively little activation of P-Rex1 and a right-shifted EC$_{50}$. EC$_{50}$= 470 nM (95% CI 160 to 13000) from 3 independent experiments. Data points correspond to the mean ± SD. (**D**) Soluble Gβγ does not activate P-Rex1 in this assay. Here, 0.5 μM PIP$_3$, 0.025 mM CHAPS, and 100 nM P-Rex1 were used, and GEF activity was normalized to that of P-Rex1 alone. These experiments were performed three times each, with the exception of the 1 μM soluble Gβγ sample which was performed two times. Error bars represent standard deviation of the mean. *P≤0.05, **P≤0.005, and *****P≤0.0005 calculated using one-way ANOVA with Dunnett's multiple comparisons post-test.

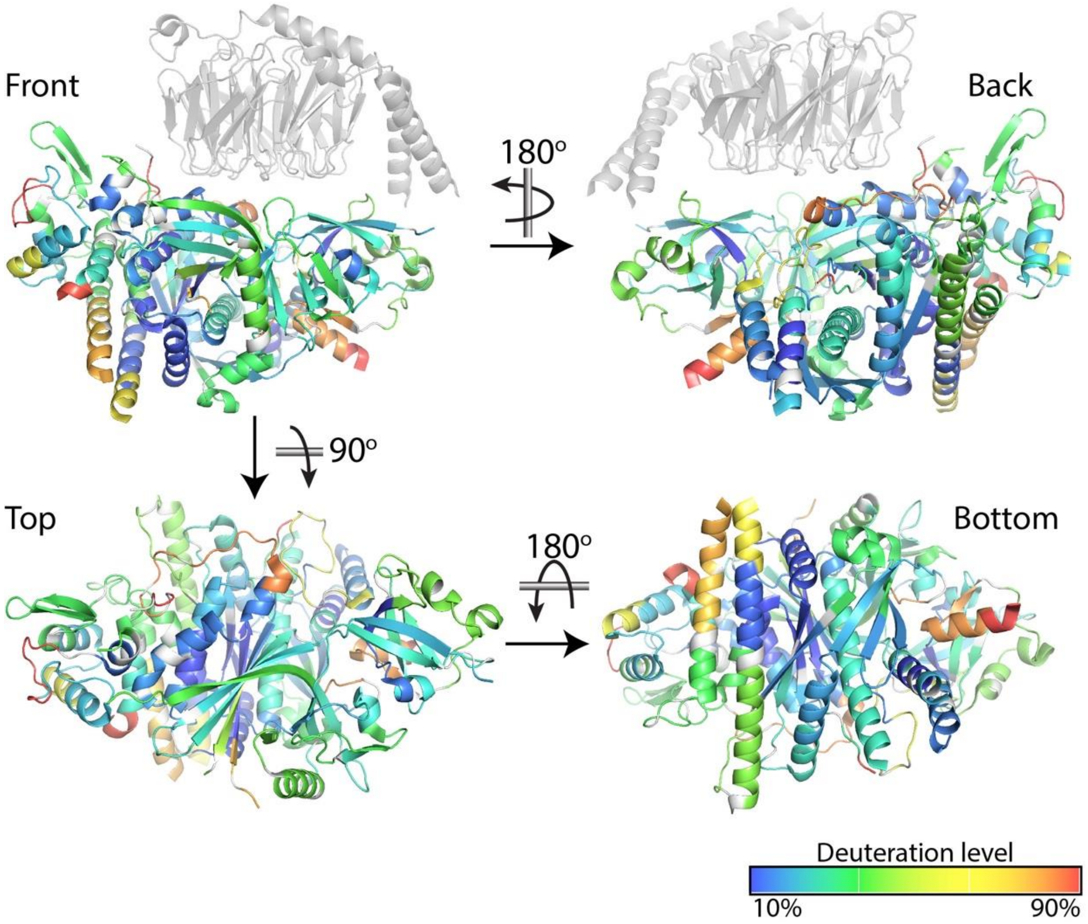

Fig. S9. HDX-MS of P-Rex1 alone. Percent deuterium exchange at the 1,000 s time point was mapped onto our cryo-EM structure of P-Rex1. Gβγ (grey) in the top images is shown only for orientation. These experiments were performed twice, and the data shown represent the average of two experiments. See also Data S1.

Table S1. Cryo-EM data collection, refinement, and validation statistics.

	Structure: P-Rex1–Gβγ<br>EMDB: EMD-20308<br>PDB: 6PCV<br>EMPIAR: EMPIAR-10285	
	Untilted	*Tilted*
**Data collection**		
Grids	Carbon Quantifoil	UltrAuFoil Quantifoil
Vitrification method	FEI Vitrobot	FEI Vitrobot
Microscope	Titan Krios	Titan Krios
Session name	18jan18c	18aug03b
Magnification	29000X	29000X
Voltage (kV)	300	300
Stage tilt (°)	0	30
Detector	K2 Summit	K2 Summit
Recording mode	Counting	Counting
Total electron exposure (e–/$Å^2$)	49.5	45.0
Number of frames	30	100
Defocus range (µm)	1 - 3	1 - 3
Pixel size (Å)	1.0	1.0
**Data processing**		
Number of micrographs	5,012	1,734
Initial particle images (no.)	600,588	304,876
Final particle images (no.)	126,265	79,334
Total particle images merged	205,599	
Symmetry	C1	
Map resolution (Å)	3.2	
**Refinement**		
Initial model used (PDB code)	3V5W, 3QIK	
Model resolution (Å)	3.2	
FSC threshold	0.143	
Map sharpening *B* factor ($Å^2$)	-122	
Model composition		
Non-hydrogen atoms	9036	
Protein residues	1155	
Ligands	0	
B factors ($Å^2$)		
Protein	83	
Ligand	N/A	
R.m.s. deviations		
Bond lengths (Å)	0.006	
Bond angles (°)	0.992	
Validation		
MolProbity score	1.71	
Clashscore	7.4	
Poor rotamers (%)	0.7	
Ramachandran plot		
Favored (%)	95.7	
Allowed (%)	4.3	
Disallowed (%)	0	

Data file S1. Ribbon maps representing HDX-MS experiments. Ribbon maps representing HDX-MS data for P-Rex1 alone, Gβγ alone, and the P-Rex1–Gβγ complex. Also shown are the changes in exchange rates that occur in P-Rex1 and Gβγ upon complex formation. These results represent an average of two experiments from two biological replicates.

Data file S2. Kinetic data for HDX-MS experiments. Kinetic data for the HDX-MS experiments showing per peptide exchange rates for P-Rex1 in the presence and absence of Gβγ. Graphs are shown in the first tab, and all values are shown in the second tab along with error values in the third tab. These results represent an average of two experiments from two biological replicates.
