# Supplementary material for: Cryo–electron microscopy structure and analysis of the P-Rex1–Gβγ signaling scaffold
Source: Sci Adv. 2019 Oct 16;5(10):eaax8855. doi: 10.1126/sciadv.aax8855 (PMC6795519; doi:10.1126/sciadv.aax8855)
Supplement: Data file S1 [file aax8855_Data_file_S1.pdf]

Ribbon Map of P-Rex1 (% deuteration)

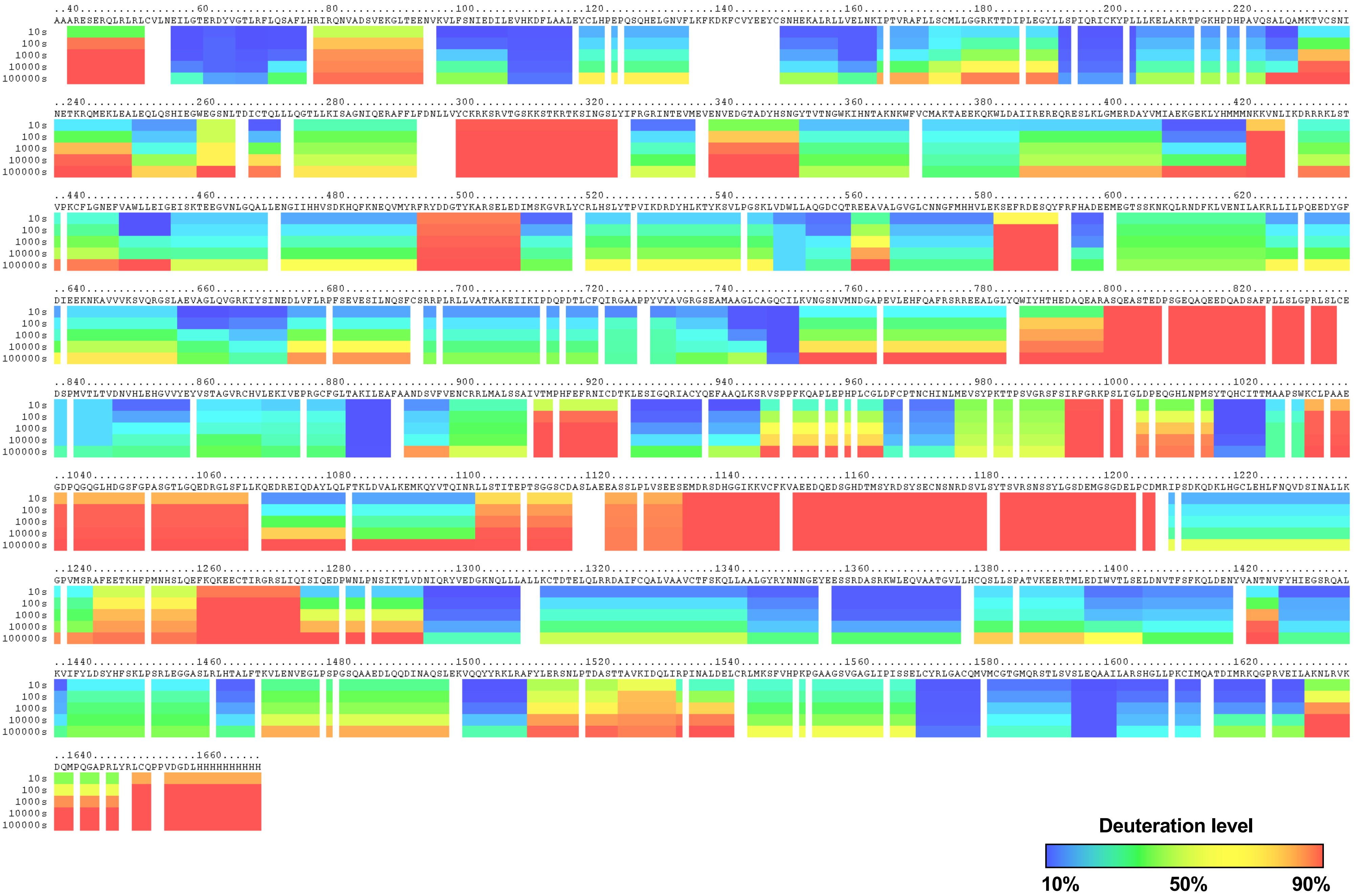

Ribbon Map of P-Rex1 in Presence of Gβγ (% deuteration)

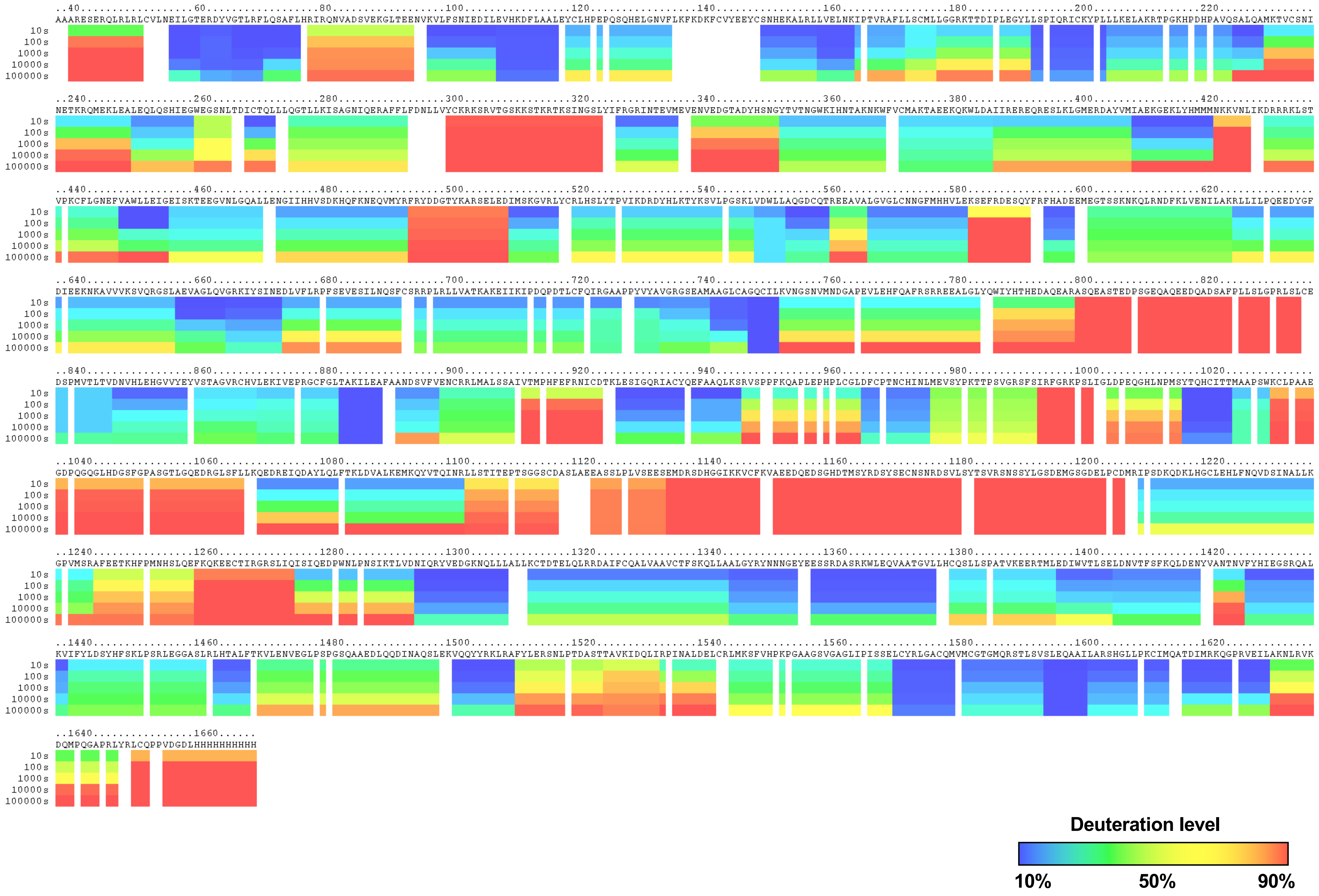

Influence of Gβγ on Exchange in P-Rex1 (% deuteration)

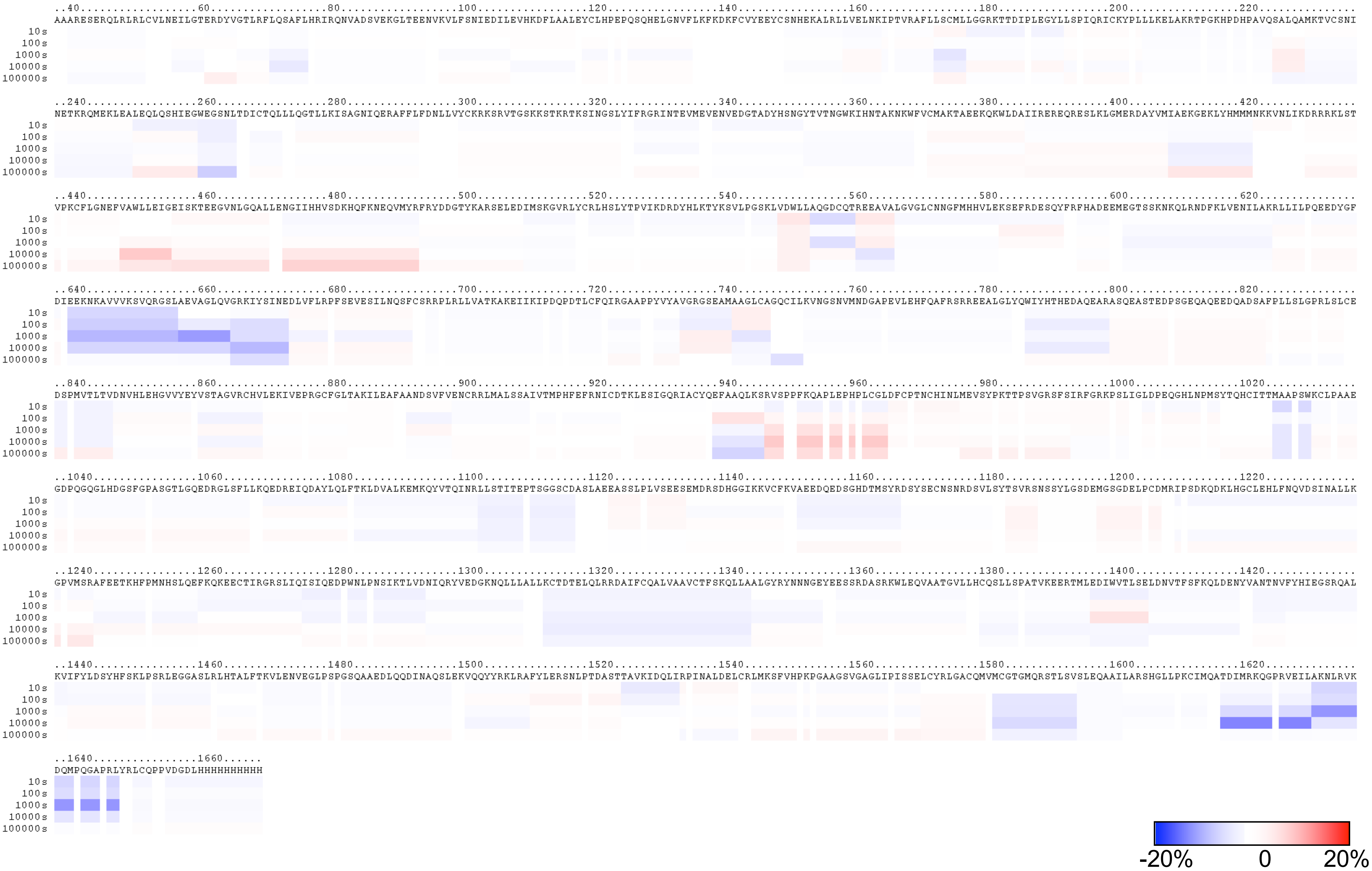

Blue indicates regions that exchange slower in presence of Gβγ. Red indicates regions that exchange faster.

Ribbon Map of Gβ (% Deuteration)

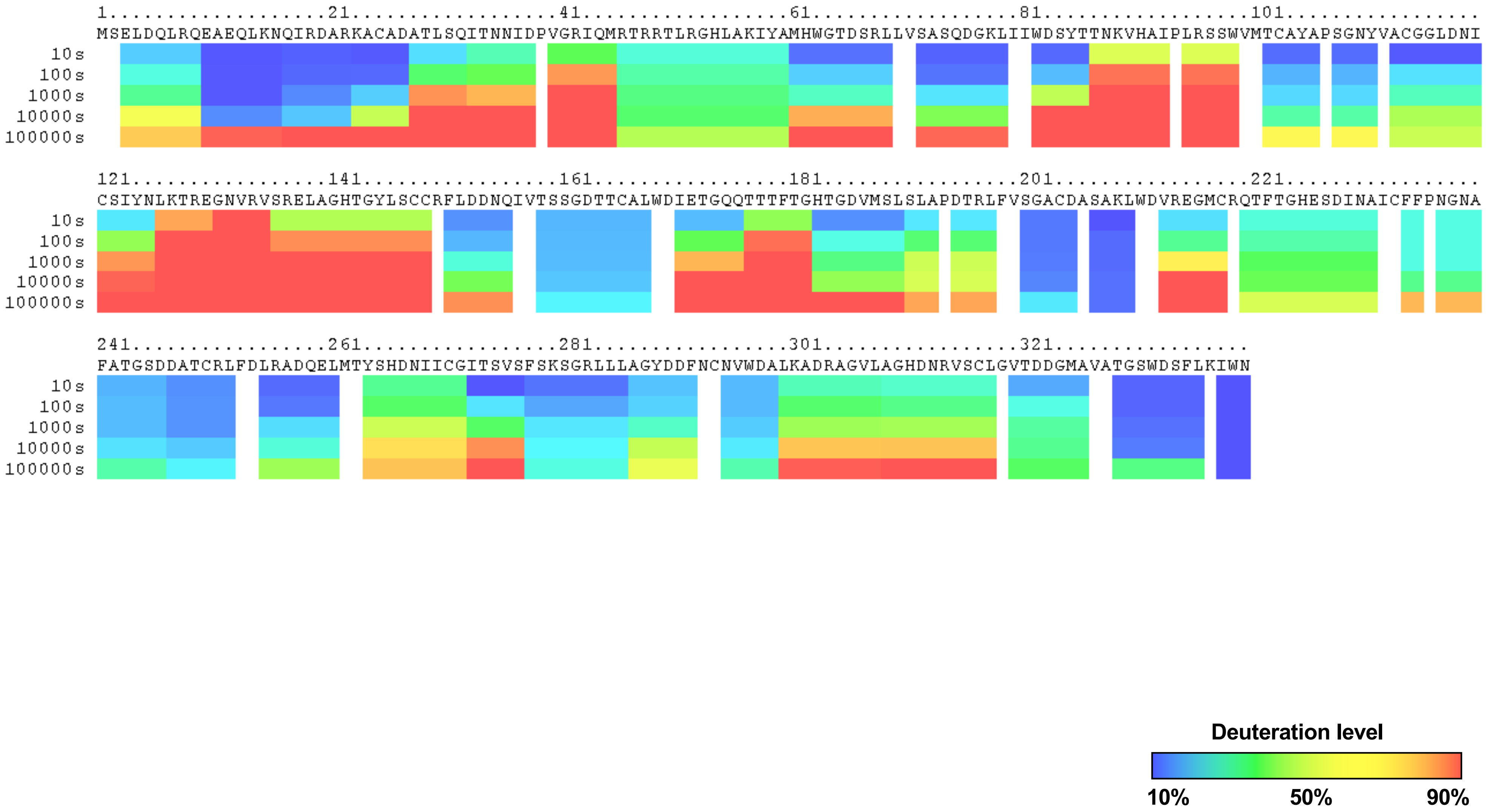

Ribbon Map of Gβ in Complex with P-Rex1 (% Deuteration)

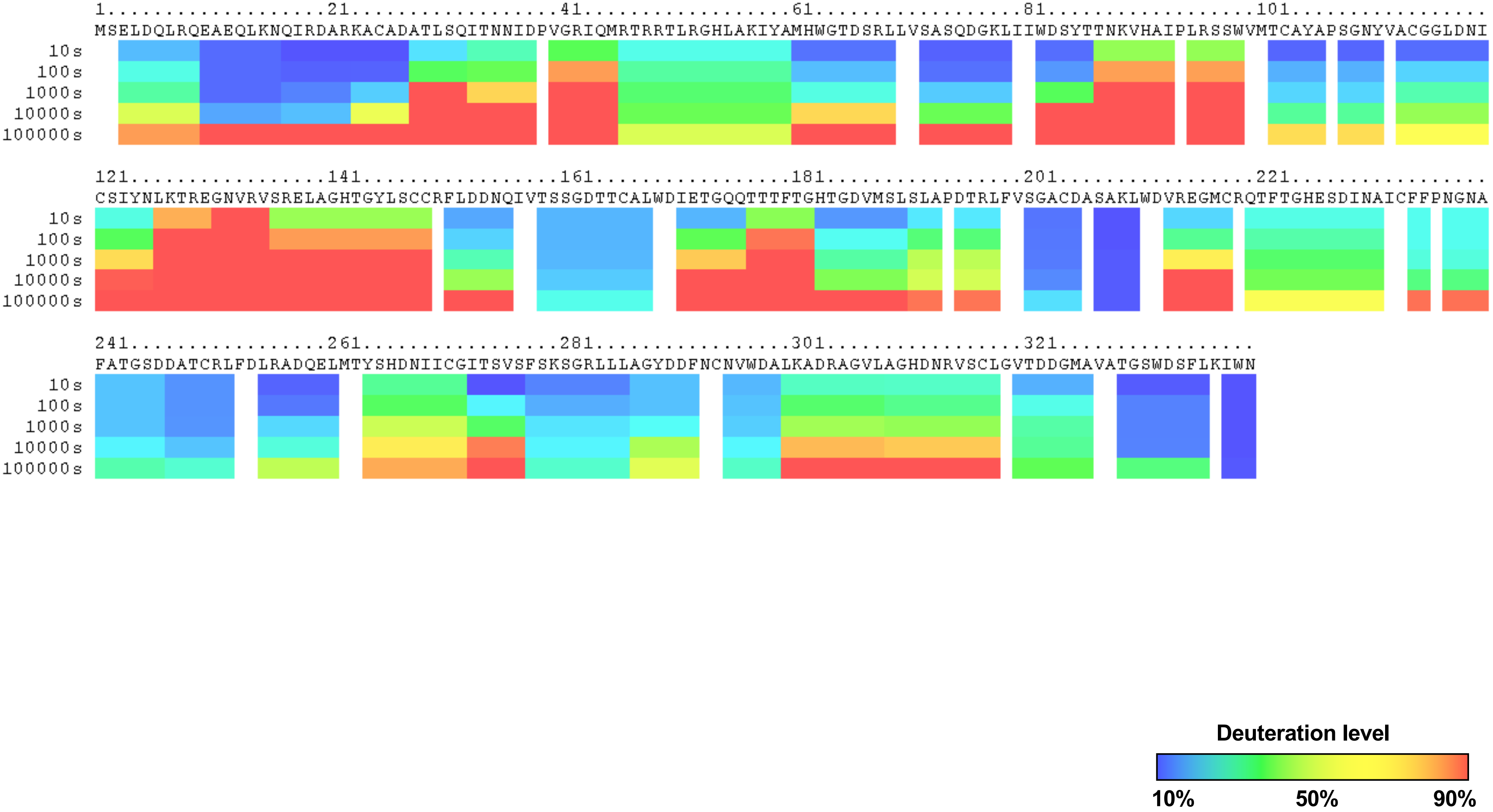

Influence of P-Rex1 on Gβ (% Deuteration)

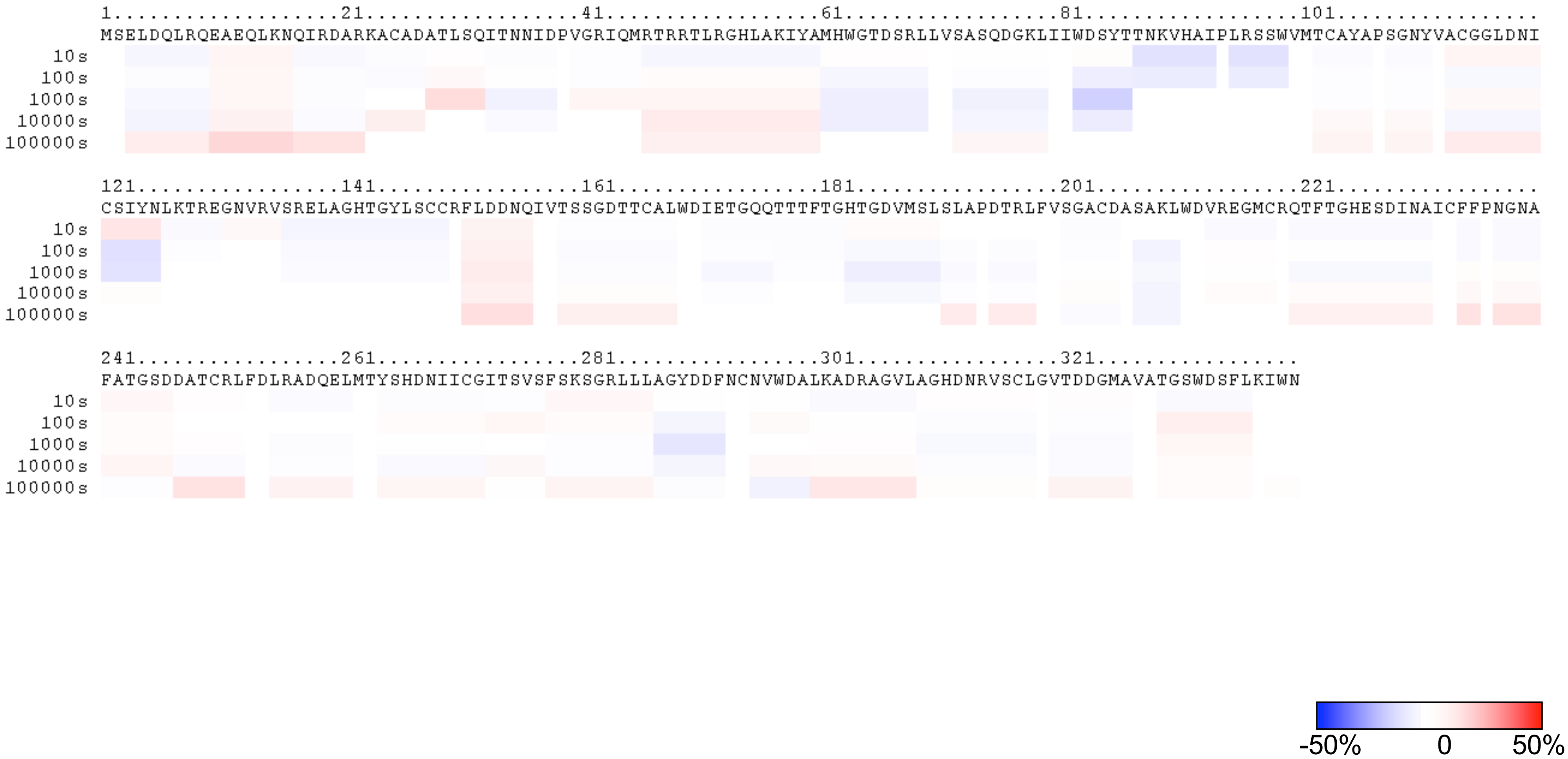

Blue indicates regions that exchange slower in presence of P-Rex1. Red indicates regions that exchange faster.

Ribbon Map of Gγ (% Deuteration)

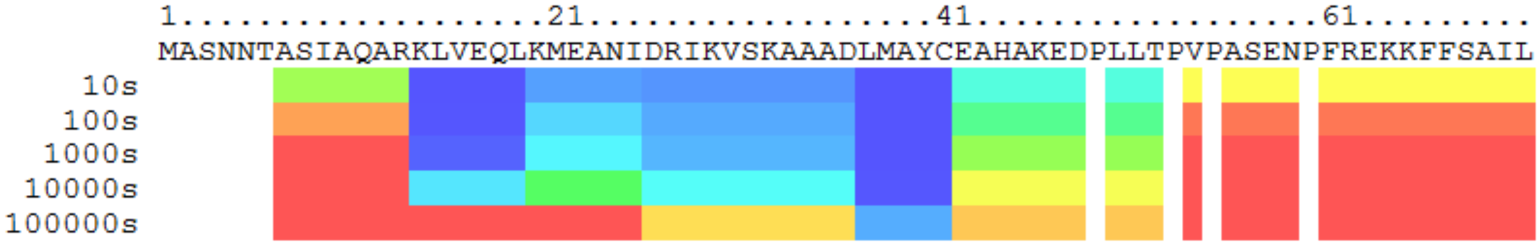

Ribbon Map of Gγ in Presence of P-Rex1 (% Deuteration)

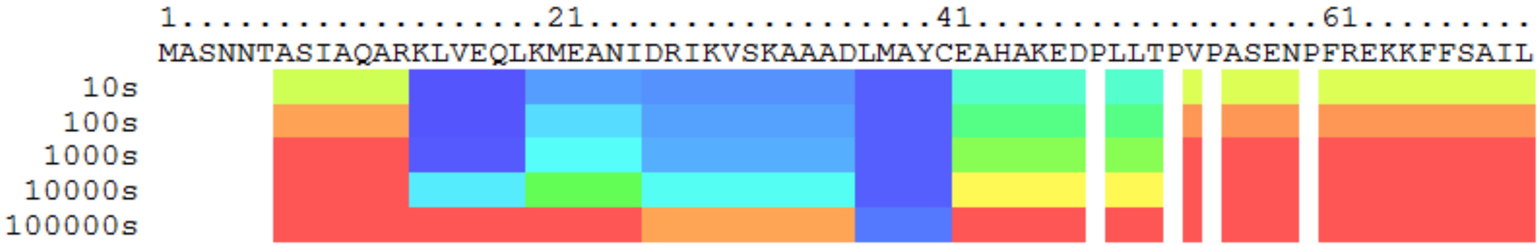

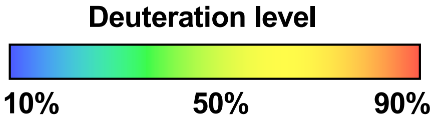

Influence of P-Rex1 on Gγ (% Deuteration)

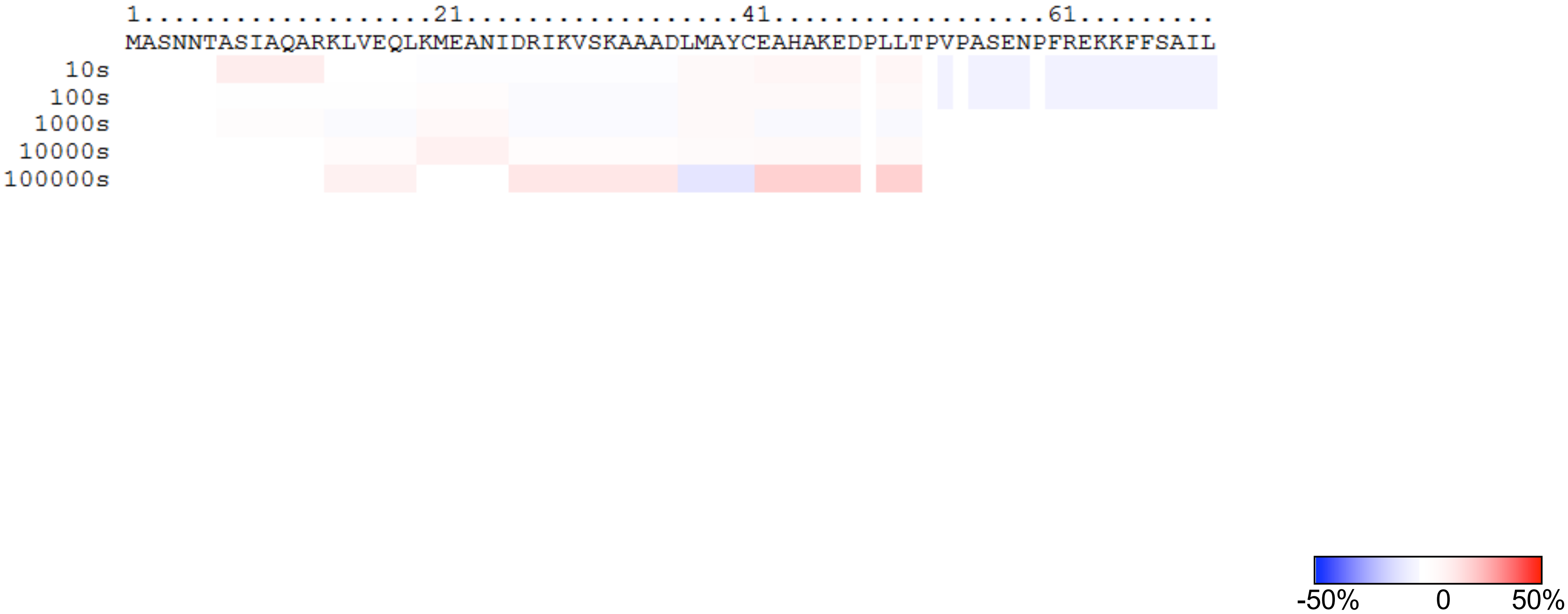

Blue indicates regions that exchange slower in presence of P-Rex1. Red indicates regions that exchange faster.
